# Characterization of X-Linked SNP genotypic variation in globally distributed human populations

**DOI:** 10.1186/gb-2010-11-1-r10

**Published:** 2010-01-28

**Authors:** Amanda M Casto, Jun Z Li, Devin Absher, Richard Myers, Sohini Ramachandran, Marcus W Feldman

**Affiliations:** 1 Department of Genetics, Stanford University, Mail Stop 5120, Stanford, California 94305, USA; 2 Department of Human Genetics, University of Michigan, 4909 Buhl Building, 1241 East Catherine St, Ann Arbor, Michigan 48109, USA; 3 HudsonAlpha Institute for Biotechnology, 601 Genome Way, Huntsville, Alabama 35806, USA; 4 Society of Fellows and Department of Organismic and Evolutionary Biology, Harvard University, 26 Oxford St, Cambridge, Massachusetts 02138, USA; 5 Department of Biological Sciences, Stanford University, Gilbert Hall 108, Stanford, California 94305, USA

## Abstract

**Background:**

The transmission pattern of the human X chromosome reduces its population size relative to the autosomes, subjects it to disproportionate influence by female demography, and leaves X-linked mutations exposed to selection in males. As a result, the analysis of X-linked genomic variation can provide insights into the influence of demography and selection on the human genome. Here we characterize the genomic variation represented by 16,297 X-linked SNPs genotyped in the CEPH human genome diversity project samples.

**Results:**

We found that X chromosomes tend to be more differentiated between human populations than autosomes, with several notable exceptions. Comparisons between genetically distant populations also showed an excess of X-linked SNPs with large allele frequency differences. Combining information about these SNPs with results from tests designed to detect selective sweeps, we identified two regions that were clear outliers from the rest of the X chromosome for haplotype structure and allele frequency distribution. We were also able to more precisely define the geographical extent of some previously described X-linked selective sweeps.

**Conclusions:**

The relationship between male and female demographic histories is likely to be complex as evidence supporting different conclusions can be found in the same dataset. Although demography may have contributed to the excess of SNPs with large allele frequency differences observed on the X chromosome, we believe that selection is at least partially responsible. Finally, our results reveal the geographical complexities of selective sweeps on the X chromosome and argue for the use of diverse populations in studies of selection.

## Abstract 2

An analysis of X-linked genetic variation in human populations provides insights into population structure and demographic patterns.

## Background

In humans, females typically carry two X chromosomes while males are haploid for almost all X-linked loci, complementing their one X chromosome with the smaller Y chromosome. This relatively small alteration to the standard of simple diploidy followed by all 22 autosomes has profound consequences for X-linked markers relative to their autosomal counterparts. Even under conditions of gender equality with respect to migration and population size, the smaller effective population size of the X chromosome means that drift may have a more profound influence upon it compared to the autosomes. Some repercussions of this are suggested by the results of Rosenberg *et al*. [1] and Ramachandran *et al*. [2], who observed that X chromosomes are generally more differentiated among human populations than are autosomes. On a worldwide scale, drift has been invoked to explain why approximately 15% of the genetic variation observed at X chromosomal single nucleotide polymorphisms (SNPs) is between populations for the 51 Centre D'etude du Polymorphism Humaine-Human Genome Diversity Project (CEPH-HGDP) populations while that figure is only 10% for the autosomes [3]. This observation raises the possibility that X-linked markers may be superior to autosomal ones for distinguishing closely related populations. In addition, each X chromosome spends two-thirds of its time carried by a female. This means that X-linked markers are disproportionately influenced by female demography, making them useful for detecting differences in the demographies of the two genders. Indeed, many recent studies have found evidence on the X chromosome for skewed female to male population size and migration rate ratios [4-6], suggesting that such differences may be the norm rather than the exception in human history.

Just as the interaction between demographic factors and genetic variation is special for the X chromosome, so too is the interaction between selective forces and X-linked genetic variation. For the autosomes, a recessive mutation must become sufficiently common to be present in homozygotes before selection can act upon it; this is not the case for the X chromosome, where recessive mutations are always exposed to selection in males. Consequently, given otherwise equal conditions, recessive beneficial mutations arising on the X chromosome are more likely to go to fixation than those arising on the autosomes, while recessive, deleterious mutations are more likely to be lost [7]. The X chromosome's haploid state in males and its smaller overall effective population size also mean that selection-driven fixation or loss of non-neutral X-linked alleles proceeds more rapidly than comparable processes on the autosomes, regardless of the initial frequency of the selected allele [8]. While it remains unclear how important recessive, non-neutral mutations are to human adaptation and to evolution in general, there is some evidence that positive selection acting on recessive, beneficial mutations has been important in shaping patterns of X-linked genetic variation in humans [9].

Given the special features of the X chromosome and of its interactions with the forces that influence human genetic variation, the analysis of patterns of X-linked genetic variation both independently and in comparison to autosomal patterns has the potential to reveal features of large genome-wide genotypic datasets that cannot be detected using autosomal markers alone. Here we use a number of methods to characterize the data represented by the approximately 16,000 X-linked SNPs typed as part of a genome-wide panel in the 51 globally distributed CEPH-HGDP populations. We begin by examining the population structure underlying variation on the X chromosome. We then use Fst values and pairwise allele frequency differences to examine population differentiation and explore what the results of these analyses indicate about past demographic patterns. Finally, we scan the X chromosome for haplotype structure consistent with the influence of selection. We finish by discussing two regions we identified as being clear outliers from the rest of the chromosome with respect to SNP allele frequency distribution and linkage disequilibrium patterns.

## Results and discussion

### Data

The dataset described previously by Li *et al. *[3] consists of 656,995 biallelic SNPs genotyped in 938 individuals from 51 populations (in this study we consider all Bantu individuals as one population and all Han Chinese individuals as one population); 16,297 of these SNPs are located within the non-pseudoautosomal region of the X chromosome. As the CEPH-HGDP sample set includes 383 females and 615 males, this dataset contains information from 1,261 X chromosomes. The non-pseudoautosomal region of the X chromosome consists of approximately 148 Mb of genome sequence, yielding a marker density of about 22 SNPs per 200 kb. This is about half the marker density of the autosomal SNPs in this dataset (reported by Pickrell *et al. *[10] to be 40/200 kb), which is expected given that a tag SNP strategy was used to select markers for the Illumnia 650K chip and that the average recombination rate on the X chromosome is about 60% of the average autosomal rate [11]. The genotypes were phased using the program fastPHASE [12]; for the X chromosome, known haplotypes from male chromosomes were also used in phasing the female chromosomes.

### Population structure

**Figure 1:**
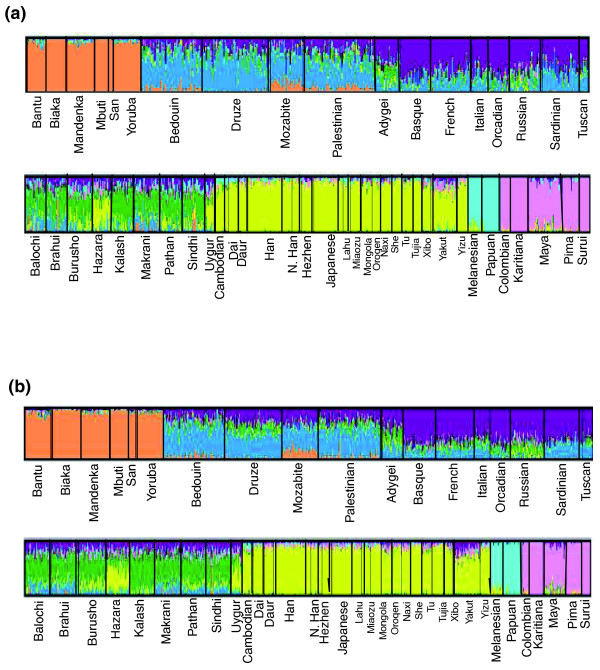
**Structure of the CEPH-HGDP Populations as estimated using *frappe***. Figures drawn using *Distruct *[45]. **(a) **Population structure estimated using 16,297 X chromosome SNP genotypes with K = 7. **(b) **Population structure estimated using 19,632 chromosome 16 SNP genotypes with K = 7.

Given the X chromosome's disproportionate sensitivity to female demography, it is possible that X-linked genomic variation has a different underlying population structure than autosomal variation. To investigate this, we analyzed the X chromosome data with *frappe *[13], a maximum likelihood based method that establishes K ancestry groups based on allele frequency patterns and then assigns each individual K percentages that correspond to his or her proportional membership in each group. As about two-thirds of the individuals in our sample are haploid for the X chromosome while one-third are diploid, we ran *frappe *on individual X chromosomes rather than on individuals. The results of this analysis with K = 7 are shown in Figure 1a. The X chromosomes are partitioned into seven clusters that correspond to the seven major continental cohorts - Africa, the Middle East, Europe, Central Asia, East Asia, Oceania, and America - represented in the CEPH-HGDP sample set. (In contrast, the data contained in 20 X-linked microsatellites was only able to resolve the CEPH-HGDP samples into 5 distinct groups [2]). These are the same 7 groups that were observed when *frappe *was run on 640,698 autosomal markers [3]. The major difference between these previous autosomal results and Figure 1a is the failure of the Eurasian X chromosomes to cleanly separate into Middle Eastern, European, and Central Asian groups. While most Middle Eastern, European, and Central Asian X chromosomes have their largest contributions from their respective continents of origin, most also have sizable contributions from the other two Eurasian continents. This suggests a lack of clear genetic distinction between chromosomes originating from these three continents. Nonetheless, the X chromosome still carries sufficient genetic information to reveal certain details of population structure that were previously noted for the large autosomal dataset. For instance, in both figures the Adygei have significant European and Central Asian contributions, the Hazara and Uygur have primarily East Asian ancestry, and a handful of Sindhi, Makrani, Brahui, and Balochi individuals have sizable African contributions. Next, to assess whether the differences we observed between our X chromosome *frappe *results and the Li *et al. *[3] autosomal results were due to the number of markers used in each analysis, we ran *frappe *on just the 19,632 markers found on chromosome 16. As with the X chromosome, the analysis was conducted using haploid chromosomes as opposed to diploid individuals. The results of the *frappe *run for K = 7 are shown in Figure 1b. Overall, the results for chromosome 16 appear very similar to those for the X chromosome. There are some minor differences between the two figures, particularly in the way some admixed populations are partitioned among the seven groups (note, for instance, the larger European component in the Yakuts and the larger Middle Eastern component in the Adygei for the autosomes). However, these differences may be artifacts of the failure of both datasets (the X chromosome and chromosome 16) to cleanly separate into the three Eurasian continental groups rather than robust differences in the population structure of autosomal and X-linked SNP genotypic variation. For completeness, we also ran *frappe *on diploid individuals. We did this first for the X chromosome by running *frappe *on all CEPH-HGDP females plus additional 'pseudofemales' created by randomly pairing two male X chromosomes from the same population. We then ran *frappe *on diploid individuals for chromosome 16 using the same number of females and 'pseudofemales'. This time 'pseudofemales' were created by randomly selecting one chromosome 16 from each male and then pairing these chromosomes within populations. The results of these analyses are shown in Additional file 1 and are quite similar to the results from running *frappe *on individual chromosomes. Also, to ensure that our choice of chromosome 16 to represent the autosomes did not bias our results, we ran *frappe *on individual chromosomes for chromosome 17. The results are largely the same (Additional file 1), except that we observe less resolution between Middle Eastern and European chromosomes for chromosome 17. We conclude from this analysis that there are no major differences in the population structure suggested for the CEPH-HGDP populations by approximately 16,000 X-linked SNPs and a similar number of autosomal SNPs.

### AMOVA

We carried out an AMOVA analysis on all X chromosome and autosomal markers using the same population and continental groupings as Ramachandran *et al*. [2] and Rosenberg *et al*. [1] (Additional file 2). We evaluated the genotypes here as haplotypic data of known phase so that all genotyped X chromosomes could be used. The results are shown in Table 1. These two previous studies demonstrated that, for microsatellite genotypes in the CEPH-HGDP, the within-population variance component is smaller for X chromosome markers than for autosomal markers for all of the various population groupings under consideration. This observation has been attributed to increased drift caused by the smaller effective population size of the X chromosome relative to the autosomes. Interestingly, while we observed the same trend for most population groupings with our SNP dataset, some notable differences were apparent. The within-population variance components for X-linked markers are indeed smaller than those for autosomal markers for the World, Eurasian, African, Oceanic, and American groupings. However, for the individual continents of the Eurasian landmass, the within-population variance components for X-linked and autosomal markers are nearly the same; for the Middle Eastern populations, the confidence intervals of the two values overlap (it should be noted, however, that the X chromosome value is smaller and that the failure of the confidence intervals to separate could be due to lack of power in the test) and for the East Asian populations, the autosomal value is actually lower than the X-linked value (the confidence intervals of the two values do not overlap for East Asians). To assess the robustness of these findings, we carried out a second AMOVA analysis for the same population groupings, evaluating the data as diploid genotypes. To use as much of the dataset as possible in this analysis, we again created 'pseudofemales' by pairing up random male X chromosomes within populations. We then randomly selected a single chromosome 16 for male samples and paired these in the same fashion so that the population sample sizes would be equal for the two marker types. For this analysis, the within-population variance component for East Asia is again smaller for autosomal markers (here represented by chromosome 16) than for X-linked SNPs (although in this case the confidence intervals of the two values overlap). This is contrary to the expectation for gender-neutral demography that, in populations of finite size, the proportion of X-linked variance occurring within populations should be smaller than that for autosomal markers and could be explained by the existence of effectively more females than males (or a higher female migration rate) in some parts of Eurasia. Such gender asymmetric demographies can be restricted in time and geographical space, which may explain why the same pattern was not evident in the microsatellite AMOVA analyses of the CEPH-HGDP populations; microsatellites have a much faster mutation rate than SNPs and so are likely to be less informative about events that occurred in the more distant past. In contrast to the studies of Rosenberg *et al*. [1] and Ramachandran *et al*. [2], a study by Segurel *et al*. [14] using microsatellite markers did find evidence for a higher female effective population size (and a higher female migration rate) in Central Asia, although it should be noted that these findings specifically applied to patrilineal herding populations and not to a more general sample set, such as the CEPH-HGDP populations.

**Table 1:** AMOVA results for 14 groupings of the Human Genome Diversity Project populations

			Variance components (95% confidence intervals)					
			
			X Chromosome			Autosomes		
				
Samples	Number of regions	Number of populations	Within populations	Among populations within regions	Among regions	Within populations	Among populations within regions	Among regions
World	1	51	86.23 (86.08,86.36)	13.77 (13.64,13.92)		90.17 (90.15,90.19)	9.83 (9.81,9.85)	
World	5	51	81.35 (81.16,81.53)	2.99 (2.96,3.02)	15.66 (15.47,15.85)	86.60 (86.58,86.63)	2.50 (2.50,2.50)	10.90 (10.88,10.92)
World	7	51	84.52 (84.36,84.68)	2.44 (2.41,2.47)	13.04 (12.88,13.20)	88.98 (88.97,89.00)	2.08 (2.07,2.08)	8.94 (8.92,8.96)
Africa	1	6	92.92 (92.78,93.05)	7.08 (6.95,7.22)		95.63 (95.62,95.65)	4.37 (4.35,4.38)	
Hunter-Gatherers	1	3	88.37 (88.04,88.70)	11.63 (11.30,11.96)		93.41 (93.38,93.44)	6.59 (6.56,6.62)	
Agriculturists	1	3	99.09 (99.01,99.18)	0.91 (0.82,0.99)		99.06 (99.05,99.07)	0.94 (0.93,0.95)	
Eurasia	1	21	97.08 (97.04,97.12)	2.92 (2.88,2.96)		97.64 (97.63,97.64)	2.36 (2.36,2.37)	
Eurasia	3	21	96.55 (96.50,96.61)	1.58 (1.55,1.61)	1.86 (1.82,1.91)	97.25 (97.25,97.26)	1.43 (1.43,1.43)	1.32 (1.31,1.32)
Middle East	1	4	98.44 (98.39,98.49)	1.56 (1.51,1.61)		98.45 (98.44,98.45)	1.55 (1.55,1.56)	
Europe	1	8	98.84 (98.79,98.89)	1.16 (1.11,1.21)		98.92 (98.91,98.92)	1.08 (1.08,1.09)	
Central Asia	1	9	98.08 (98.02,98.13)	1.92 (1.87,1.98)		98.33 (98.33,98.34)	1.67 (1.66,1.67)	
East Asia	1	17	98.45 (98.40,98.51)	1.55 (1.49,1.60)		98.40 (98.40,98.41)	1.60 (1.59,1.60)	
Oceania	1	2	88.94 (88.55,89.33)	11.06 (10.67,11.45)		90.30 (90.25,90.35)	9.70 (9.65,9.75)	
America	1	5	89.04 (88.85,89.24)	10.96 (10.76,11.15)		90.66 (90.63,90.68)	9.34 (9.32,9.37)	

### Pairwise allele frequency differences

The AMOVA scores calculated above provide an estimate of how differentiated populations within a particular continental or supracontinental group are from one another. We would expect, though, that the effects of drift and selection would be most pronounced between two genetically distant populations, given the time that these forces have had to affect allele frequencies in each population independently. Because of this, we selected three pairs of distantly related populations - Yoruba-Han, Yoruba-French, and French-Han, and calculated for each autosomal and X-linked marker the pairwise allele frequency difference (termed 'delta' or 'δ' by Shriver *et al*. [15]) for each pair. We found that the average delta value was higher for X-linked than for autosomal markers for all three pairs. We also noted that the distributions of X-linked delta values all have a longer 'tail' region than the autosomal distribution for the same population pair. To examine these tail regions more closely, we tallied the number of SNPs for which delta exceeded 0.9 (hereafter referred to as high-delta SNPs) for each population pair (Table 2). On the X chromosome, there were no SNPs for which delta > 0.9 in the French-Han comparison, so for this population pair we tallied the number of X-linked SNPs for which delta > 0.8. High-delta SNPs on the autosomes and on the X chromosome often occur in clusters, with each cluster presumably representing a single event, be it drift or selection. To gain a rough estimate of the number of such events, we divided the autosomes into 13,395 200-kb regions; each region containing at least one high-delta SNP was deemed a high-delta region. While some high-delta regions did contain only one high-delta SNP, many contained multiple high-delta SNPs. We carried out the same process with the X chromosome, where there were a total of 744 200-kb regions.

**Table 2:** Results of the delta analysis for three population comparisons

			Yoruba-Han		Yoruba-French		French-Han			
							
	Total		Delta > 0.9		Delta > 0.9		Delta > 0.9		Delta > 0.8	
					
	Autosome	X	Autosome	X	Autosome	X	Autosome	X	Autosome	X
Total SNPs	640,698	16,297	265	159	62	25	6	0	107	25
Regions	13,395	372	174	43	52	15	4	0	57	11

Overall, we observed that there were proportionally more high-delta SNPs on the X chromosome than on the autosomes for population pairs with one African and one non-African population (25 out of 16,297 compared to 62 out of 640,698 and 159 out of 16,297 compared to 265 out of 640,698 for the Yoruba-French and Yoruba-Han comparisons, respectively; Table 2). For the French-Han comparison, this excess of high-delta SNPs on the X chromosome was not observed. This apparent disparity between the three population pairs could be explained by a female-specific bottleneck during the out of Africa migrations as recently suggested by Keinan *et al*. [5]. When there are equal numbers of males and females, the X chromosome is more heavily influenced by drift than the autosomes due to its smaller population size; this effect is exaggerated when there are fewer females than males. But is drift alone sufficient to explain the excess X-linked high-delta SNPs found for the Yoruba-Han and Yoruba-French pairs? To address this question, we utilized an equation developed by Segurel *et al. *[14] that expresses the expected relationship between X-linked and autosomal Fst values in terms of N_f_/N, the female proportion of the effective population size, and m_f_/m, the female proportion of the total migration rate. This equation was derived from known relationships between Fst values and male and female migration rates and effective population sizes under the infinite island model with populations of equal and constant size. We used the equation to obtain expected delta values for the X-linked SNPs from the observed autosomal delta values. If autosomal and X-linked markers differed collectively only by the relative effects of drift, transformed autosomal delta values (expected X-linked values) should not differ statistically from observed X-linked values. We applied this transformation to our three lists of autosomal delta values varying N_f_/N and m_f_/m from 0.01 to 0.99. As the female portion of the effective population size and migration rate in humans has likely varied widely across time and geographical distance, we wanted to test across all possible values of N_f_/N and m_f_/m, including 'N_f_/N, m_f_/m' pairs where N_f_/N < 0.5, as such pairs represent female specific bottlenecks (that is, more than half of the population is male).

**Figure 2:**
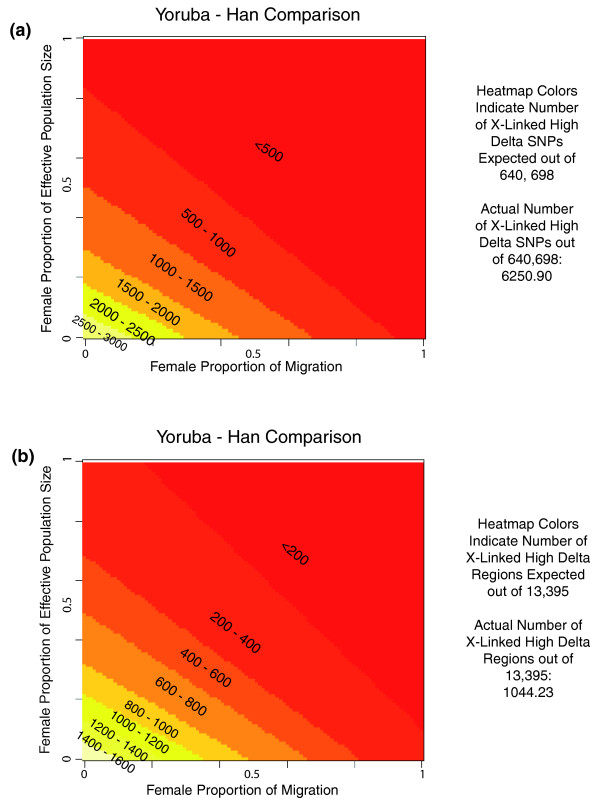
**Number of high-delta SNPs and regions represented by TA/EX values for the Yoruba-Han comparison**. **(a) **The female proportion of the effective population size and the female proportion of migration were both varied over a range from 0.01 to 0.99. For each of the 9,800 possible pairs of these values, a list of TA/EX values was produced from the observed autosomal delta values for the Yoruba-Han. The color at a given point on the grid represents the number of high-delta SNPs out of 640,698 total SNPs found in this list. **(b) **The same as in (a), except that the color at a given point on the grid signifies the number of high-delta regions out of 13,395 total regions represented by each list of TA/EX delta values.

Having transformed each of our three lists of autosomal delta values for all possible pairs of N_f_/N and m_f_/m such that 0.01 ≤ N_f_/N, m_f_/m ≤ 0.99, we tabulated the number of high-delta SNPs in each of the resulting lists of transformed autosomal/expected X chromosome (hereafter referred to as TA/EX) values (for the French-Han pair, we tabulated the number of SNPs with delta exceeding 0.8). The results are shown in Figure 2 for the Yoruba-Han population pair and in Additional file 3 for the Yoruba-French and French-Han pairs. (In the transformation, the values of N_f_/N and m_f_/m are combined into a single term, given by (1 + m_f_/m)/(2 - N_f_/N). Because N_f_/N and m_f_/m are combined this way, there are multiple N_f_/N, m_f_/m value pairs that produce the same TA/EX delta values. This feature of the Segurel *et al*. [14] transformation creates the diagonal bands of color in Figure 2 and Additional file 3). We see that for the TA/EX delta values to contain the same number of high-delta SNPs (or SNPs where delta exceeds 0.8 for the French-Han pair) as were observed on the X chromosome, extreme values must generally be used for both N_f_/N and m_f_/m (for the Yoruba-French pair, N_f_/N must be less than 0.08 and m_f_/m must be less than 0.05, and for the Yoruba-Han pair, there are, in fact, no such values). Having transformed our three lists of autosomal delta values for all pairs of N_f_/N and m_f_/m such that 0.01 ≤ N_f_/N, m_f_/m ≤ 0.99 and re-tabulated the number of high-delta SNPs in each (alternatively, the number of SNPs with delta exceeding 0.8 for the French-Han pair), we also assigned these SNPs to one of the 13,395 autosomal regions. The resulting tallies of high-delta regions represented by each list of TA/EX delta values are shown in Figure 2 for the Yoruba-Han pair and in Additional file 3 for the Yoruba-French and French-Han pair. Again we see that for the TA/EX delta values to contain the same number of high-delta regions (or regions containing a SNP where delta exceeds 0.8 for the French-Han pair) as were observed on the X chromosome, low values must generally be used for both N_f_/N and m_f_/m (for the Yoruba-French pair, N_f_/N must be less than 0.52 and m_f_/m must be less than 0.36, while for the Yoruba-Han pair, N_f_/N must be less than 0.29 and m_f_/m must be less than 0.18).

**Figure 3:**
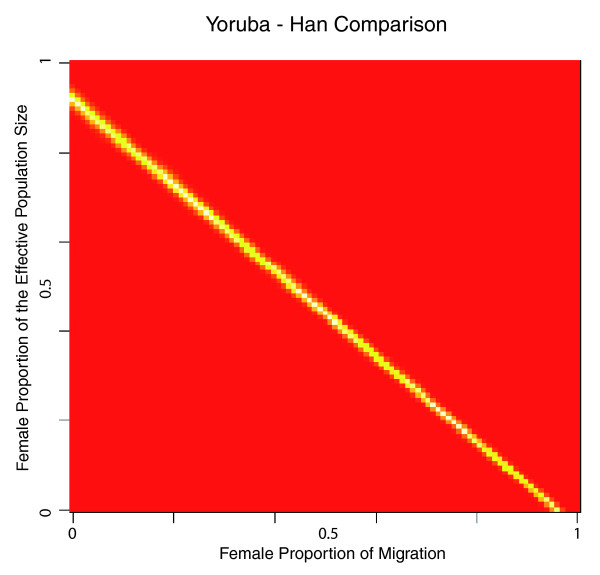
**Comparison of TA/EX and observed X-linked delta values**. The female proportion of the effective population size and the female proportion of migration were both varied over a range from 0.01 to 0.99. For each of the 9,800 possible pairs of these values, a list of TA/EX delta values was produced from the observed autosomal delta values for the Yoruba-Han comparison. This list of TA/EX values was compared to the list of observed X-linked delta values using a two-sided Wilcoxon test. The color at a given point represents the resulting *P*-value of this comparison. Red locations on the grid represent N_f_/N, m_f_/m value-pairs that produce sets of TA/EX delta values that significantly differ from the observed X-linked values; white and yellow regions on the grid represent N_f_/N, m_f_/m value-pairs that produce TA/EX values that do not significantly differ from the observed X-linked values.

It is possible, of course, that we observe a large number of X-linked high-delta SNPs because the populations under study here were characterized by low values for N_f_/N and m_f_/m (due to, for instance, population bottlenecks; Additional file 4). To assess which values of N_f_/N and m_f_/m are most consistent with the distributions of autosomal and X-linked delta values that we observe, we again varied N_f_/N and m_f_/m from 0.01 to 0.99. We then compared each resulting list of TA/EX delta values to the observed X-linked values using a two-sided Wilcoxon test. The results of this analysis are shown in Figure 3 for the Yoruba-Han pair and Additional file 5 for the Yoruba-French and French-Han pairs. By comparing the results shown in Figures 2 and 3, one can see that the overall distributions of TA/EX and observed X-linked delta values are most similar for sets of TA/EX delta values with proportionally fewer high-delta SNPs than were observed on the X chromosome. This indicates that while there are N_f_/N, m_f_/m value pairs that produce TA/EX delta values with proportionally similar numbers of high-delta SNPs compared to what was observed for the X chromosome, these N_f_/N, m_f_/m pairs are not consistent with the distributions of autosomal and X-linked delta values that we observe. Overall, our results here suggest that even after accounting for the differential effects of drift on the X chromosome and the autosomes, there have been proportionally more events affecting the X chromosome that cause significant allele frequency changes resulting in high-delta SNPs. The above analyses were also carried out using pairwise Fst values in place of delta with similar results (Additional files 6 and 7); an excess of high Fst SNPs and regions was observed on the X chromosome for the Yoruba-Han and Yoruba-French pairs and an excess of SNPs with Fst > 0.8 was observed on the X chromosome for the French-Han pair.

Previous studies (Coop *et al*. [16]; Barreiro *et al*. [17]) have also noted that a disproportionate number of high-delta and high-Fst SNPs lie within coding regions. We did not necessarily expect to make the same observation for the X chromosome, since the hitchhiking of non-coding variants on selected genic alleles is likely to be more common on the X chromosome. Indeed, while 32% (5,213 out of 16,297) of our X-linked markers are in genes, we found that only 26.4% of all Yoruba-Han high-delta SNPs were located within genes on the X chromosome. However, after removing a large cluster of high-delta SNPs (one that contained 68 high-delta SNPs, including 65 non-coding ones) from consideration, this percentage jumped to 44.4%. SNPs with large allele frequency differences in the other two population comparisons were also commonly found in genes. Of the SNPs with delta > 0.8 in the French-Han comparison, 52% were genic, as were 76% of the high-delta SNPs from the Yoruba-French comparison (Table 3). In general, we observed that bins of X-linked high-delta SNPs were enriched for genic SNPs, while bins of X-linked SNPs with delta values closer to 0 were not (Additional file 8). This observation could be explained by an excess of genic SNPs with a minor allele frequency ≤ 0.1. However, we detected no such excess but noted that high-delta SNPs simply occur more frequently among genic SNPs where the minor allele frequency ≤ 0.1 than among non-genic SNPs meeting the same criterion. These findings suggest that at least some of the high-delta regions we have identified on the X chromosome have undergone selective sweeps, as selection is more likely to have targeted coding variants than non-coding variants; drift acting alone would be expected to influence coding and non-coding variation equally.

**Table 3:** Characteristics of X-chromosomal high-delta SNPs

	Yoruba-Han	Yoruba-French	French-Han	
			
	Delta > 0.9	Delta > 0.9	Delta > 0.9	Delta > 0.8
Genic SNPs	43/159	19/25	0/0	13/25
High derived frequency (in second population)	133/159	22/25	0/0	13/23

For each of the X chromosomal high-delta SNPs, we determined which allele was derived and which ancestral using information from two chimpanzees that were genotyped along with the HGDP samples in Li *et al. *[3] and information from the NCBI website [18]. We were able to determine the ancestral state for the majority of the autosomal and X-linked high-delta SNPs. For the Yoruba-French comparison, 3 out of 25 (12%) high-delta SNPs had a high derived frequency in the Yorubans, and for the Yoruba-Han comparison, 26 out of 159 (16.4%) high-delta SNPs had a high derived frequency in the Yorubans. For the autosomes, we found that only 5 out of 58 (8.6%) high-delta SNPs had a high derived allele frequency in Africa in the Yoruba-French comparison; that figure was 18 out of 247 (7.3%) in the Yoruba-Han comparison (Table 3). The percentage of X-linked high-delta SNPs with high derived allele frequency in Africa significantly exceeds (chi square test, *P *< 0.001) that for the autosomes in the Yoruba-Han comparison; this could be explained by a higher incidence of hitchhiking on the X chromosome compared to the autosomes. An alternative, and intriguing, possibility is that the X chromosome has been affected by a disproportionate number of selective sweeps or drift events (for example, bottlenecks) involving derived alleles in Africa. Looking back to our identification of genic and non-genic high-delta SNPs, we found some evidence that selection may indeed be a player in this observation. Recall that for the Yoruba-Han comparison (when we excluded the one exceptional high-delta region, 65.5 to 67 Mb), 44.4% of all high-delta SNPs were in genic regions. If we take only those high-delta SNPs that have high derived allele frequency in Africa, this increases to 50%. Similarly, all three high-delta SNPs from the Yoruba-French comparison with high derived frequency in the Yorubans are found in genes.

### Tests of selection (iHS, CLR, XP-EHH)

**Figure 4:**
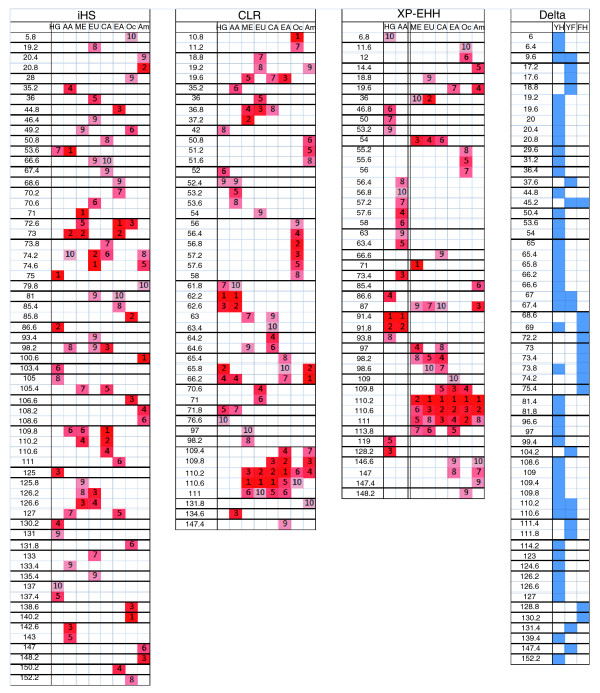
**Top ten regions for iHS, CLR, and XP-EHH scores and all high-delta regions**. The numbers on the left side of each figure represent the beginning position of a region in megabases. Each chromosomal region is 400-kb in length. The letters across the top of each figure represent the eight continental regions: HG, hunter-gatherer; AA, African agriculturist; ME, Middle East; EU, Europe; CA, Central Asia; EA, East Asia; Oc, Oceania; Am, America. In the delta figure, the column labels represent the three population comparisons: YH, Yoruba-Han; YF, Yoruba-French; FH, French-Han. For iHS, CLR, and XP-EHH, each small column contains the numbers 1 to 10 denoting the top ten X-chromosomal regions for each particular test and continental group with the intensity of the color corresponding to the rank. For XP-EHH, the double line separating the African agriculturist column from the Middle East column signifies that the XP-EHH scores represented on the left side of the lines were calculated using East Asians as the reference sample while the scores represented on the right side of the lines were calculated using African agriculturists as the reference sample (Additional file 9). For delta, each blue shaded region contains either a high-delta SNP for the Yoruba-Han or Yoruba-French comparison, or a SNP with delta > 0.8 for the French-Han comparison.

To investigate the relative importance of drift and selection in creating large interpopulation allele frequency differences on the X chromosome, we wanted to ascertain whether X-linked high-delta SNPs tend to occur in regions where the haplotype structure is consistent with the past influence of selection. We subjected our dataset to three tests - integrated haplotype score (iHS), combined likelihood ratio (CLR), and cross population extended haplotype homozogysity (XP-EHH) - that were designed to produce high scores in chromosomal regions that have been involved in selective sweeps. Although we will refer to iHS, CLR, and XP-EHH as 'tests of selection', it should be remembered that these tests identify regions where selection may have influenced allele frequencies or haplotype patterns; demographic forces are always a possible explanation for one high iHS, CLR, or XP-EHH score or an entire set of elevated scores, including the scores we report below. CLR and XP-EHH are most sensitive to nearly completed sweeps [19,20], while iHS is useful for detecting on-going, partial sweeps [21]. iHS, CLR, and XP-EHH were run on each of the eight continental groups - African agriculturists, African hunter-gatherers, Middle Eastern, European, Central Asian, East Asian, Oceanian, and American - individually (CLR and XP-EHH were also calculated for selected individual populations; Additional file 9). Then, following recommendations from previous work [10], we divided the X chromosome into 372 400-kb regions and, for each continental group, calculated one iHS, CLR, and XP-EHH score for each region using the raw scores from that region (see Materials and methods for details). In order to briefly characterize the results of these calculations, we selected the top ten regions with respect to test value for each test in each continental group and displayed them in Figure 4. As can be seen, the distribution of top regions across the X chromosome and the relationship between the lists of top regions across continents is rather different for iHS than for CLR and XP-EHH. Top iHS regions are rarely consecutive for any given continent and the same region is typically not highlighted for more than one continent. The observation that high iHS signals are often not shared across geographical regions has been commented on previously [10]. As top iHS signals do not tend to cluster in adjacent chromosomal regions, and as iHS results do not generally overlap with CLR and XP-EHH scores (since iHS alone detects sweeps in progress), we suggest that it is difficult to use iHS by itself to detect targets of past selection; here we use iHS results only as additional, complementary evidence to argue for past selection at a given site on the X chromosome. Unlike iHS, sharing of top signals between certain continents is noticeable with CLR and XP-EHH. Despite a deep phylogenetic split between the two groups (see Figure 1B in [3]), the top CLR and XP-EHH signals for African agriculturists and hunter-gatherers cluster in the same two regions, 62.2 to 63 Mb and 91.4 to 92.2 Mb, respectively. Neither of these regions produces top CLR or XP-EHH signals for any of the other continental groups. The sharing of these top signals despite long-standing genetic separation could suggest a genomic response to a selective pressure produced by a common African environment. Eurasian groups also tend to produce top CLR and XP-EHH scores in the same X-chromosomal regions. This is not surprising given their close genetic relationship, and was also observed for autosomal CLR and XP-EHH scores [10]. However, for several consecutive chromosomal regions, 109.4 to 111.4 Mb, top CLR and XP-EHH signals appear not only in Eurasia, but also in East Asia, Oceania, and America. Specifically, this segment of the X chromosome produces the top two CLR scores for Europe, Central Asia, and East Asia, the top two XP-EHH scores for Central Asia, East Asia, Oceania, and America, and top iHSs in the Middle East and Central Asia; it will be discussed in more depth in a subsequent section.

Figure 4 displays all of the 61 400-kb X chromosome regions that contain either a high-delta SNP in the Yoruba-French or Yoruba-Han comparison, or a SNP with a delta value > 0.8 in the French-Han comparison. We found that 31 of these regions also produced a top iHS, CLR, or XP-EHH score for at least one continent. As iHS and XP-EHH are based on haplotype frequencies, scores for these two tests and delta values are not expected to be totally independent of one another (although the overall correlation between delta values and test scores seems to be fairly low; for example, the Pearson correlation between Yoruba-Han delta values and raw XP-EHH scores in East Asia is only 0.1764). However, the presence of a high Fst SNP in a genomic region producing a high XP-EHH score has previously been taken as evidence that the region is a true target of selection rather than a false positive [20]. It is also interesting to note that these 31 regions were not a random sample of the 61 high-delta regions. There were 4 clusters along the X chromosome - 18.8 to 20.8 Mb, 65 to 67.4 Mb, 72.2 to 74.2 Mb, and 108.6 to 110.6 Mb - of 4 or more consecutive high-delta regions, and of the 23 individual 400-kb regions in these clusters, 20 produced top iHS, XP-EHH, or CLR scores. Conversely, of the 19 high-delta regions that occurred in isolation (that is, they were not bordered on either side by another high-delta region), only 3 produced a top iHS, CLR, or XP-EHH score. It seems then that X-linked high-delta SNPs, particularly those that occur in clusters along the chromosome, tend to be found in chromosomal regions where iHS, CLR, and XP-EHH suggest that the haplotype structure is consistent with selection at that site.

**Figure 5:**
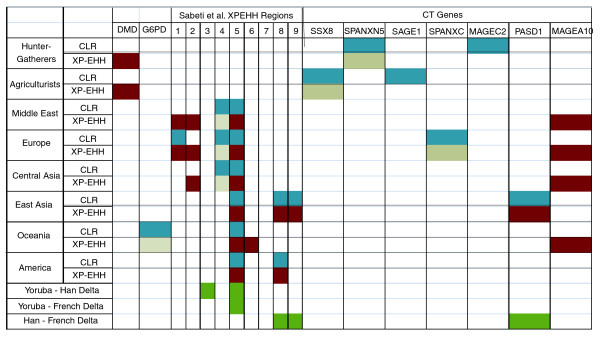
**Previously implicated X-linked selection targets with elevated CLR, XP-EHH, or delta scores**. Each row represents a particular delta comparison or a particular test as labeled. Each column represents a X-chromosomal region or locus that previous research has identified as being under selection. Colored boxes designate regions where elevated test scores were observed (see text and Materials and methods for details); blue boxes represent elevated CLR scores, red boxes represent elevated XP-EHH scores, and green boxes represent elevated delta values. Gray boxes denote regions where there are no scores for the corresponding test.

Finally, we wanted to evaluate whether our results support the conclusions of any of the previous studies of selection on the X chromosome. As most of these studies have been conducted with a few populations at the most, we were particularly interested in whether there was evidence for selection at previously implicated chromosomal sites but in populations not previously studied. Several X-linked genes have been suggested as selection targets in earlier studies (Table 4). Many of them belong to a class known as cancer/testis, or CT genes. While the molecular functions of many CT gene products are not well understood, most are believed to play a role in spermatogenesis [22]. The remaining genes (listed in Table 4 as 'other genes') were investigated in single gene studies and are associated with a particular Mendelian trait of interest. The numbered regions listed in Table 4 were the top X-linked regions based on XP-EHH score identified as part of a full genome survey [20]. These XP-EHH scores were calculated using 3 million SNPs typed in the HapMap samples. To determine whether our work supported the hypothesis of selection acting on these regions and the genes discussed above, we tabulated and averaged all the CLR scores, XP-EHH scores, and delta values occurring within a given gene or region. We then compared this average score to averages of all other sets of consecutive scores of the same size (for instance, if there are *x *CLR scores in region A, we calculated the average CLR score for all other *x*-sized sets of consecutive CLR scores). If the average score for our region or gene of interest was higher than the averages of 95% of the other such regions, we considered this evidence of selection on this chromosomal region. The results of this analysis are outlined in Figure 5. We saw no evidence of selection (by our criteria) for most of the non-CT genes from our literature survey. The only two exceptions for this were DMD, which produced high XP-EHH scores in both African groups, and G6PD, which produced high CLR scores in Oceania. Several CT genes did contain high CLR and/or XP-EHH scores, most notably MAGEA10, which contained high XP-EHH scores in all three Eurasian continents and Oceania, although these were not accompanied by significantly elevated CLR scores. We also found some evidence for selection in seven of the regions outlined by Sabeti *et al*. [20]. Importantly, for six out of seven of these regions, evidence for selection was found in continental groups not represented in the HapMap samples, allowing us to more fully define the geographic extent of these putative selective events.

**Table 4:** Chromosome position of X-linked genes and regions found to be under selection in previous studies

Gene/region	Position	
CT genes		
*MAGEB2*	30,143,601	30,148,127
*MAGEB3*	30,158,474	30,165,528
*FTHL17*	30,999,279	31,000,091
*SSX1*	47,999,279	48,011,823
*PAGE1*	49,339,008	49,347,307
*SSX8*	52,668,710	52,679,723
*SPANX-N5*	52,841,911	52,843,113
*CPXCR1*	87,888,882	87,896,441
*CXorf48*	134,118,127	134,133,417
*SAGE1*	134,803,451	134,822,886
*SPANXF1*	139,912,422	139,925,542
*SPANXC*	140,163,262	140,164,312
*SPANXA1*	140,499,462	140,506,565
*MAGEC3*	140,753,768	140,813,284
*MAGEC2*	141,117,796	141,129,742
*SPANX-N4*	141,941,370	141,949,732
*SPANX-N3*	142,424,230	142,432,973
*SPANX-N2*	142,622,721	142,632,182
*FMR1NB*	146,870,541	146,915,876
*PASD1*	150,482,663	150,595,867
*MAGEA4*	150,831,652	150,844,298
*MAGEA5*	151,033,182	151,037,100
*MAGEA10*	151,053,564	151,057,681
		
Other genes		
*ACE2*	15,489,077	15,530,199
*DMD*	31,047,266	33,267,647
*MAOA*	43,400,353	43,491,012
*CD40L*	135,558,002	135,570,215
*F9*	138,440,561	138,473,283
*OPN1LW*	151,877,661	151,892,305
*G6PD*	153,412,800	153,428,981
		
XPEHH results [20]		
Region 1	18,811,880	19,138,487
Region 2	35,759,035	35,939,638
Region 3	36,476,826	36,521,901
Region 4	37,069,665	37,555,024
Region 5	109,767,056	111,117,626
Region 6	113,291,719	113,296,616
Region 7	141,796,760	141,804,088
Region 8	147,341,578	147,421,230
Region 9	150,287,808	150,488,109

### Chromosomal regions of interest

In evaluating our results, we identified two regions that were clear outliers from the rest of the chromosome. The genic and SNP content of these regions are discussed in detail below along with the evidence that led us to identify them as outliers.

#### 65 to 67 Mb

**Figure 6:**
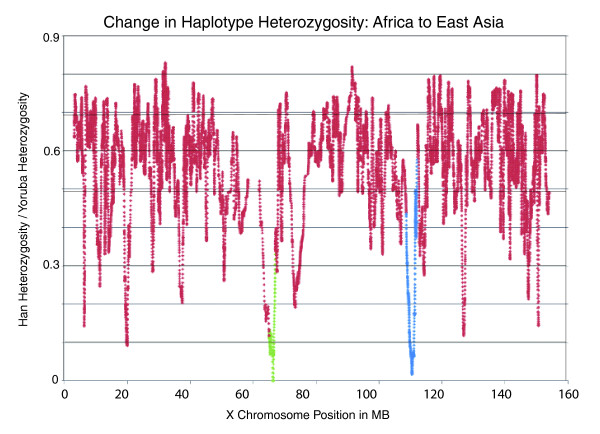
**Change in haplotype heterozygosity between African agriculturists and East Asians**. All sets of five consecutive X-linked SNPs were assembled into haplotypes. Haplotype heterozygosity was then calculated for all haplotypes in the Yoruba and the Han. The abscissa represents the chromosomal position of the center SNP while the ordinate gives the value of the Han heterozygosity as a proportion of the Yoruba heterozygosity (Han heterozygosity/Yoruba heterozygosity). The green data points represent haplotypes centered between 65 and 67 Mb and the blue data points represent haplotypes centered between 108.6 and 112.2 Mb.

This region was first detected by the delta analysis; in the Yoruba-Han comparison, 77 high-delta SNPs were found here. All 67 of the high-delta SNPs found between 65.7 and 67 Mb had a delta score of 1, while the remaining 10 SNPs had a delta score less than 1. Although the clustering of such a large number of high-delta SNPs in one region could be partially explained by an extremely low recombination rate, we found that rates in this region (0.9 CM/Mb on average for 65 to 66 Mb and 0.7 CM/Mb for 66 to 67 Mb) were only about one-half to three-quarters that of the X chromosome average of 1.2 CM/Mb, and were higher than those of neighboring regions [23]. The large number of high-delta SNPs in this region was accompanied by a near total loss of haplotype heterozygosity in East Asia (Figure 6). Our CLR analysis also highlighted this region of the chromosome as one of interest; it produced high CLR values for the East Asian and the American populations as well as the African populations. Interestingly, high iHSs were observed between 65 and 67 Mb for Europe and Central Asia but not for Africa, East Asia, or the Americas.

**Figure 7:**
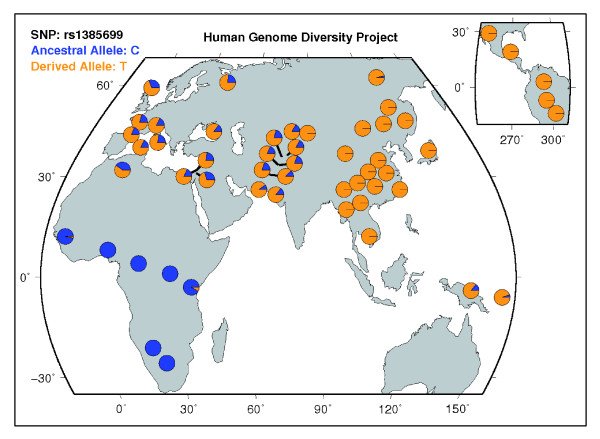
**Frequency spectra of rs1385699 in CEPH-HGDP populations**. rs1385699 is a non-synonymous SNP located in *EDA2R*. This image was downloaded from the UCSC Genome Browser Website [47].

Next, we investigated the genic content of this region. Our search yielded four genes, one of which, *EDA2R*, encodes the ectodysplasin 2A receptor and is closely related to the autosomally encoded ectodysplasin 1A receptor (*EDAR*). Indeed, while each receptor is thought to have its own unique, non-redundant function, they bind two ligands, ectodysplasin 2A and ectodysplasin 1A, that differ from one another by only two amino acids and both serve to activate the NF-KB pathway [24]. *EDAR*, which has recently become of interest to human population geneticists as a target of positive selection in humans [25], harbors a non-synonymous mutation that is fixed in East Asian and American populations. The altered protein that results from this mutation and its effects on the NF-KB pathway are now thought to be an important determinant of East Asian hair thickness [25]. *EDA2R *also contains a non-synonymous mutation with a high allele frequency difference between Africans and East Asians. Indeed, this SNP (rs1385699) was one of the high-delta SNPs identified in this region; the derived allele frequency is 0% in Yorubans and 100% in the Han Chinese, although unlike the *EDAR *SNP, this derived allele is present at high frequencies in Eurasia (Figure 7). rs1385699 has also recently been associated with a hair-related phenotype; an association study of Sardinian men linked the polymorphism to androgenic alopecia, commonly known as male pattern baldness [26]. The close relationship between *EDAR *and *EDA2R *and the obvious similarities in the allele frequency spectra of their non-synonymous SNPs make it tempting to theorize that these two polymorphisms could have become targets of selection due to their affects on a single favored phenotype. However, the molecular consequences of the rs1385699 mutation are far from clear. Unlike the *EDAR *mutation, which alters an amino acid in the receptor's binding site, rs1385699 changes an amino acid in one of three cysteine rich regions of *EDA2R*, which are not involved in ligand binding [20,26]. Further work is clearly necessary to understand the relationship, if any, between these two mutations. It should also be noted that *EDA2R *is not the only gene in this region that could be a target of selection. Immediately upstream of *EDA2R *is hephaestin (*HEPH*), which encodes a product essential for the proper uptake of iron from the diet [27]. Downstream from *EDA2R *lies the androgen receptor (*AR*), which, with its role in sexual development and subsequently in fertility and reproductive ability, is theoretically attractive as a target of selection [28]. Moreover, the gene is known to harbor a number of polymorphisms that, like rs1385699, have been associated with androgenic alopecia [29]. The androgenic alopecia risk alleles for these polymorphisms are contained within a single haplotype block that may have been subject to selection in Europe [30], although we found that the largest CLR values occurred somewhat upstream of this area. Indeed, it should be noted that the vast majority (62 out of 77) of the high-delta SNPs that first attracted our attention to this region lie not in the *AR *or *EDA2R *genes, but in the long intergenic region that separates these two loci. Hillmer *et al*. [30] also noted that this intergenic region shows high levels of linkage disequilibrium and produces high mean Fst scores among the HapMap populations. The accumulated evidence suggests that this region may contain an important regulatory element.

#### 108.6 to 112.2 Mb

This area of the X chromosome was a clear outlier in the tests of selection. High iHS, CLR, and XP-EHH scores were observed between 108.6 and 112.2 Mb for all non-African continental groups. The highest scores were generally observed in the 800-kb region from 110.2 to 111 Mb. This area also contained a total of 13 high-delta SNPs from the Yoruba-Han comparison and 9 high-delta SNPs from the Yoruba-French comparison. As with the 65 to 67 Mb region, there is near total loss of haplotype heterozygosity in East Asia in this region (Figure 6). In examining this region of the X chromosome for genic targets of selection, we focused our attention from 110.2 to 111 Mb because of the distribution of iHS, CLR, and XP-EHH scores and the location of the aforementioned high-delta SNPs. It was not clear which of the five genes in this region is the most likely target of a selective sweep. Of these five genes, three - *PAK3*, *DCX*, and *TRPC5 *- encode proteins that are thought to be most active in the brain, with *PAK3 *and *DCX *being particularly involved in neuronal migration [31,32]. A fourth gene, *CAPN6*, encodes a calcium-dependent cysteine protease also found in the brain and in the placenta [33], while the fifth gene, *ALG13*, has a yeast homolog active in N-glycosylation [34]. *PAK3*, *TRPC5*, and *CAPN6 *have all been implicated in certain human diseases, including Alzheimer's disease for *PAK3 *[35] and neurodegenerative disease for *CAPN6 *[36]. Several cases of X-linked mental retardation have been linked to rare variants in *PAK3 *[37] and recent research has theorized that *TRPC5 *may play a role in the pathogenesis of rheumatoid arthritis [38]. All five of these genes are known to carry at least one non-synonymous mutation, although none of them have large intercontinental allele frequency differences.

#### 62.2 to 63 Mb

High iHS, CLR, and XP-EHH scores tended to cluster on different parts of the chromosome for African and non-African populations. While the chromosomal segments described above produced high scores for the tests of selection in non-African groups, high CLR scores were observed over the interval 62.2 to 63 Mb for the two African groups. Specifically, the first and third highest CLR scores for the African hunter-gatherers occurred here along with the first and second highest CLR scores for the African agriculturists. This chromosomal region contains three genes - *SPIN4*, *LOC92249*, and *ARHGEF9*. Mutations in *ARHGEF9*, which encodes a Rho-like GTPase, are associated with epilepsy and hyperekplexia (hypersensitivity to certain external stimuli) [39]. Little is known about *SPIN4 *and *LOC92249*. However, all three genes lie between 62.6 and 63 Mb while the highest CLR scores are observed between 62.2 and 62.6 Mb, so the target of selection in this region, if any, may lie outside of a known gene.

#### 91.4 to 92.2 Mb

Another chromosomal region producing high test-of-selection scores in the African populations is 91.4 to 92.2 Mb, where the top two XP-EHH scores in both the African hunter-gatherers and agriculturists were observed. The highest XP-EHH scores in this region correspond to the SNPs found between 91.4 and 91.8 Mb, which is the location of the gene *PCH11X*, a member of the protocadherin family of cell adhesion and recognition proteins [40]. *PCH11X *has a homolog on the Y chromosome and is not subject to X inactivation. Despite this, *PCH11 *transcript levels are twice as high in females compared to males [41]. Previous studies have reported evidence of selection on particular members of the protocadherin family, including the alpha protocadherin cluster on chromosome 5 and recently *PCH11Y *[42,43].

## Conclusions

We have explored the possible impacts of both demography and selection on X-linked genetic variation. With regards to the former, we were particularly interested in investigating the possibility of male versus female demographic differences as these can be detected by comparing autosomal and X-linked data. Previous studies have found evidence for skewed gender ratios. Indeed, here we showed that evidence for asymmetries in both directions (male N_e_/female N_e _less than or greater than 1) can be found within a single dataset. Our results suggest that the picture of male versus female demography is complex and that each study addressing this question should be viewed as providing insight on a particular geographical scale and period in history rather than an absolute answer.

Of the three analyses that were potentially informative with respect to asymmetries in the demographics of the two genders (population structure using *frappe*, AMOVA, and delta analysis), we focused particularly on the results of the delta analysis. We did so because the differences between the X chromosome and the autosomes were so marked for this analysis, because these differences were robust to correction for drift, and because this feature of X chromosomal genetic variation has not previously been noted. We observed that more high-delta SNPs occurred in genes than would be expected by chance and that many high-delta SNPs occurred in regions with top iHS, CLR, or XP-EHH scores. Given these two pieces of evidence, we believe that while demographic processes and drift are important in shaping X-linked genotypic variation, the forces of selection are necessary to explain the observed excess of X-linked high-delta SNPs.

As selection is likely to have been important in shaping patterns of genetic variation on the X chromosome, we used iHS, CLR, and XP-EHH scores to identify possible targets of selection. Our objective in this was both to identify novel targets and use the diverse populations in our dataset to better define the geographical extent of previously described selective sweeps. We found that putative sweeps often encompass neighboring continents, but that the pattern is complex. Coop *et al*. [16] enumerated three major geographical distributions for selective sweeps as 'West Eurasian', 'East Asian', and 'non-African' sweeps, but we found evidence that certain subtypes exist. For instance, some non-African sweeps extend to Oceania and America, while some do not. We also found evidence for selection at several loci previously implicated as X-linked selection targets and our results show that previously described sweeps often extend outside the populations in which they were originally discovered. These findings reinforce the importance of using geographically diverse sample sets in scans for genomic targets of selection.

Finally, we highlighted two X-chromosomal regions that are outliers relative to the rest of the X chromosome with respect to SNP allele frequency distribution and haplotype structure. We believe that it is likely these loci were influenced by selection in the past. In the case of the 65 to 67 Mb region, we found a promising candidate for a target polymorphism - rs1385699, a non-synonymous SNP with known phenotypic associations and large allele frequency differences between African and East Asian populations. Overall, both regions represent interesting foci for future research into the role of selection in shaping genetic diversity on the X chromosome.

## Materials and methods

### *Frappe*

We used the program *frappe *[13] to estimate the population structure underlying 16,297 X-linked SNP genotypes. The input files for *frappe *were generated using plink [44]; each X chromosome was converted into a diploid individual by making all loci homozygous for each haploid genotype. *Frappe *was then run with a maximum iteration of 500 and a step of 100 with K set to 7. The program output was displayed as a figure using *Distruct *[45]. This process was repeated to estimate population structure for 19,632 chromosome 16 SNP genotypes.

### FST

The AMOVA analysis was carried out on the X chromosome using the program Arlequin [46]. A total of 14 CEPH-HGDP population groupings were analyzed, including 12 that were previously examined by Rosenberg *et al*. [1] and Ramachandran *et al*. [2] using microsatellite markers. We also included two additional groupings by dividing the six African populations into hunter-gatherer and agriculturist groups. The AMOVA values reported for each of the 14 groupings were calculated using all of the X-linked markers that were polymorphic within a particular group. The 95% confidence intervals were calculated from 20,000 bootstrap runs. This process was then repeated to calculate the reported AMOVA values for chromosome 16.

### Delta

We calculated the allele frequency difference, or delta, for each of the 656,995 SNPs in our dataset for three population pairs: Yoruba-Han, Yoruba-French, and Han-French. For each comparison, we selected all SNPs for which delta was greater than 0.9 and called these high-delta SNPs. Once we observed that there were no X-linked high-delta SNPs for the Han-French comparison, we enumerated all SNPs in this comparison for which delta exceeded 0.8. To determine how many of the X-linked SNPs in our dataset were in genic regions, we downloaded the chromosomal positions of known genes from the UCSC Genome Browser Website [34]. All SNPs found within annotated gene boundaries were scored as genic SNPs. The ancestral allele for some SNPs was established using genotypes for two chimpanzees that were genotyped along with the CEPH-HGDP samples on the 650K Illumina chip. For SNPs that were fixed in the two chimpanzees, the fixed allele was taken as the ancestral allele. SNPs that were either polymorphic in the chimpanzees or for which there were missing data were not assigned an ancestral allele. We then searched for SNPs without an assigned ancestral allele in the NCBI database. Ancestral allele information from this database allowed us to assign ancestral alleles to some of these remaining SNPs.

### iHS

Of the 16,297 X-chromosomal SNPs in our database, we were able to calculate raw iHSs for 11,623 to 15,532, depending on the continental group analyzed. We used an EHH cutoff value of 0.1, rather than the standard value of 0.05, in order to slightly increase the number of scores that we were able to obtain. For each continent, we then calculated the average raw iHS observed for each observed derived allele frequency. Any SNP with an iHS that differed by 2 or more from the average score for the same observed allele frequency was considered a high iHS SNP. After breaking the X chromosome up into 372 400-kb regions, we tabulated both the total number of SNPs with an iHS per region and the total number of high iHS SNPs per region. The iHS assigned to each 400-kb region is the ratio of these two values (number of high iHS SNPs: number of total SNPs with iHSs; Additional file 10).

### CLR

For each continental group, we ran the short and long arms of the X chromosome separately, using a grid size of 30,000 for the short arm and 50,000 for the long arm. All SNPs that had been assigned an ancestral allele were treated as unfolded. For each sample set, we then converted the raw CLR scores into 372 data points by assigning to each 400-kb region the average CLR score observed in that region (Additional file 10).

### XP-EHH

For each continent and each test, a chromosomal region was assigned the value of the average XP-EHH score observed within that region for that test. As with iHS, XP-EHH scores cannot be calculated for SNPs near the centromere and the chromosome ends. This left some regions near these physical boundaries with no raw XP-EHH scores for some rounds of XP-EHH testing. These regions were assigned a value of zero for that round (Additional file 10).

## Abbreviations

AR: androgen receptor; CEPH-HGDP: Centre D'etude du Polymorphism Humaine-Human Genome Diversity Project; CLR: combined likelihood ratio; CT: cancer/testis; EDAR, ectodysplasin 1A receptor; iHS: integrated haplotype score; SNP: single nucleotide polymorphism; TA/EX: transformed autosomal/expected X chromosome; XP-EHH: cross population extended haplotype homozogysity.

## Authors' contributions

JZL, DA, and RM carried out the genotyping and performed the quality control for the dataset. AMC, SR, and MWF conceived of and planned the study. AMC carried out the data analysis and wrote the manuscript with assistance from JZL, SR, and MWF.

## Supplementary Material

Additional file 1 Results of running *Frappe *on diploid individuals for chromosome 16 and the X chromosome and on individual chromosomes for chromosome 17. Click here for file

Additional file 2 Results of an AMOVA analysis of X-linked and chromosome 16 markers treated as diploid genotypes. Click here for file

Additional file 3 Number of high-delta SNPs and regions contained in various sets of TA/EX values for the Yoruba-French and French-Han population pairs. Click here for file

Additional file 4 Possible effects of a population bottleneck on N_f_/N and m_f_/m values. Click here for file

Additional file 5 Comparison of observed X-linked delta values to TA/EX values using a Wilcoxon test for the Yoruba-French and French-Han population pairs. Click here for file

Additional file 6 Number of high Fst SNPs and regions contained in various sets of TA/EX values and comparison of observed X-linked Fst values to various TA/EX Fst values using a Wilcoxon test for all three population pairs. Click here for file

Additional file 7 Results of one-sided Wilcoxon tests comparing specific sets of TA/EX Fst values to observed X-linked Fst values. Click here for file

Additional file 8 Genic enrichment (relative to the ratio of genic SNPs to all SNPs) of SNPs in different delta bins for all three population pairs. Click here for file

Additional file 9 Sample sets used for different iHS, CLR, and XP-EHH runs. Click here for file

Additional file 10 Raw iHS, CLR, and XP-EHH scores for all 372 X chromosome regions and all 8 continental groups. Click here for file
