# Supplementary material for: Characterization of X-Linked SNP genotypic variation in globally distributed human populations
Source: Genome Biol. 2010 Jan 28;11(1):R10. doi: 10.1186/gb-2010-11-1-r10 (PMC2847713; doi:10.1186/gb-2010-11-1-r10)
Supplement: Additional file 1 — Results of running Frappe on diploid individuals for chromosome 16 and the X chromosome and on individual chromosomes for chromosome 17. [file gb-2010-11-1-r10-S1.doc]

**A.**


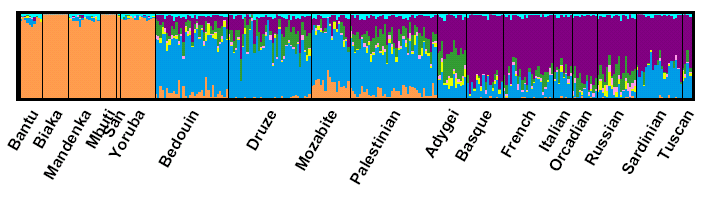


**B.**

**C.**

**Figure S1: Structure of the CEPH-HGDP Populations as estimated using *Frappe*.** Figures drawn using *Distruct* [37]. A) Population structure estimated for the 323 CEPH-HGDP females and 296 additional “pseudofemales” using the 16,297 chromosome X SNP genotypes with K = 7. *Frappe* was run on diploid individuals rather than on individual chromosomes. “Pseudofemales” were created by randomly pairing up male X chromosomes within populations to create diploid individuals. For populations with an odd number of males, one X chromosome was not used in this analysis. B) Population structure estimated for the 323 CEPH-HGDP females and 296 additional “pseudofemales” using the 19,632 chromosome 16 SNP genotypes with K = 7. Again *Frappe* was run on diploid individuals rather than on individual chromosomes. “Pseudofemales” were created by selecting a single chromosome 16 from males and randomly pairing up these chromosomes within populations to create diploid individuals. Population sample sizes were equal for the analyses in A and B. C) Population structure estimated for all CEPH-HGDP chromosomes using the 16,539 chromosome 17 SNP genotypes with K = 7. For this figure, *Frappe* was run on individual chromosomes rather than on diploid individuals. This figure is quite similar to the one in the main text created using chromosome 16 markers. The most noticeable difference is that the separation between Middle Eastern and European chromosomes is less well defined for chromosome 17 than for chromosome 16.
