# Supplementary material for: Characterization of X-Linked SNP genotypic variation in globally distributed human populations
Source: Genome Biol. 2010 Jan 28;11(1):R10. doi: 10.1186/gb-2010-11-1-r10 (PMC2847713; doi:10.1186/gb-2010-11-1-r10)
Supplement: Additional file 2 — Results of an AMOVA analysis of X-linked and chromosome 16 markers treated as diploid genotypes. [file gb-2010-11-1-r10-S2.doc]

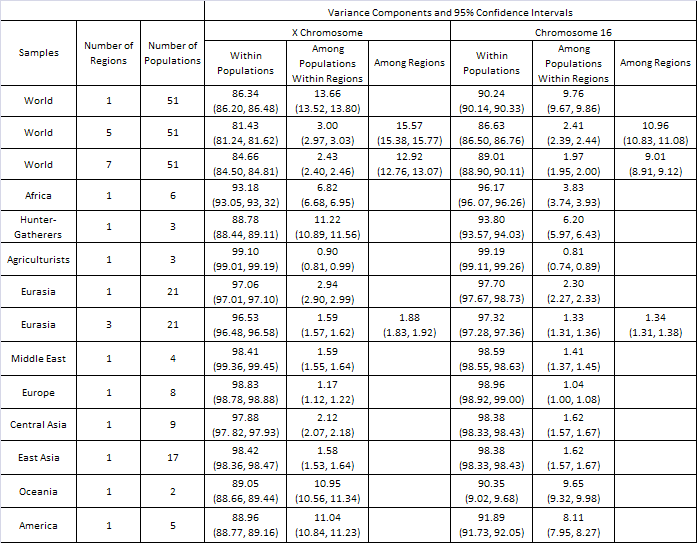


**Table S1: AMOVA Results for 14 Groupings of the CEPH-HGDP Populations.** The above table gives the results of an AMOVA analysis of the CEPH-HGDP populations divided into 14 groups. Here we treated the data as diploid genotypes and used as a sample set 323 females and 296 “pseudofemales” for both the X chromosome and chromosome 16. Again for most of the 14 groupings, the differentiation between the various included populations is greater for the X chromosome. Note, however, that like the AMOVA analysis in the main text, the between-population variance component is higher for the autosomes in East Asia, although the confidence intervals of the autosomal and X-linked values overlap in this case.
