# Supplementary material for: Characterization of X-Linked SNP genotypic variation in globally distributed human populations
Source: Genome Biol. 2010 Jan 28;11(1):R10. doi: 10.1186/gb-2010-11-1-r10 (PMC2847713; doi:10.1186/gb-2010-11-1-r10)
Supplement: Additional file 3 — Number of high-delta SNPs and regions contained in various sets of TA/EX values for the Yoruba-French and French-Han population pairs. [file gb-2010-11-1-r10-S3.doc]

**A.**

**B.**

**C.**

**D.**

**Figure S2: Number of High Delta SNPs and Regions represented by TA/EX Values for the Yoruba-French and French-Han Comparisons.** A) The female proportion of the effective population size and the female proportion of migration were both varied over a range from 0.01 to 0.99. For each of the 9,800 possible pairs of these values, a list of TA/EX delta values was produced from the observed autosomal delta values for the Yoruba-French population pair. The color at a given point on the grid represents the number of high delta SNPs out of 640,698 total SNPs found in this list. B) The same as in A, except that the observed French-Han autosomal delta values were used as input for the transformation and the color at a given point on the grid represents the number of SNPs with delta > 0.8 out of 640,698 total SNPs found in this list. C) The same as in A, except that the color at a given point on the grid signifies the number of high delta regions out of 13,395 total regions represented by each list of TA/EX delta values. D) The same as in B, except that the color at a given point on the grid signifies the number of regions containing a SNP with delta > 0.8 out of 13,395 total regions represented by each list of TA/EX delta values.
