# Supplementary material for: Characterization of X-Linked SNP genotypic variation in globally distributed human populations
Source: Genome Biol. 2010 Jan 28;11(1):R10. doi: 10.1186/gb-2010-11-1-r10 (PMC2847713; doi:10.1186/gb-2010-11-1-r10)
Supplement: Additional file 4 — Possible effects of a population bottleneck on Nf/N and mf/m values. [file gb-2010-11-1-r10-S4.doc]

**Note S1**

**The Effect of Population Bottlenecks on N_f_/N and m_f_/m**

Bottlenecks affect X-linked and autosomal variation differently. Specifically, immediately following recovery from a bottleneck, the ratio of X-linked to autosomal genetic diversity (expected to be 0.75 under conditions of constant population size) may drop as low as 0.3 (depending on the narrowness of the bottleneck) [47]. Following a bottleneck, this ratio will increase, because X-linked diversity rebounds more quickly than does autosomal diversity. Segurel et al. [14] recognized that in their model (which is also the model we use to calculate TA/EX values from observed autosomal delta values) a decrease in the ratio of X-linked to autosomal diversity would be reflected as a decrease in the female fraction of the effective population size. Our results show that having a proportionally small number of females in the population is not by itself sufficient to explain the excess of X-linked high delta SNPs that we observe (a small relative number of females *and* a proportionally low rate of female migration would both be required; see Figure 2 and Figure S2). In addition, if a bottleneck were responsible for the large number of X-linked high delta SNPs that we observe, we would expect the distribution of X-linked delta values to reflect a reduction in female population fraction (since a bottleneck would be expected to affect all loci). However, a comparison of observed X-linked and autosomal delta values suggests that the data are consistent with female population fractions around 0.5 (given female migration proportions of around 0.5) (Figure 3 and Figure S3). Interestingly, for a female migration proportion of 0.5, Yoruba-Han and Yoruba-French delta values are most consistent with a female population fraction of slightly less than 0.5 (approximately 0.45). This small bias toward a reduced female population fraction for the two African/non-African population comparisons could reflect a small reduction in the ratio of X-linked to autosomal diversity and so could be a lingering result of the out-of-Africa bottleneck.
