# Supplementary material for: Characterization of X-Linked SNP genotypic variation in globally distributed human populations
Source: Genome Biol. 2010 Jan 28;11(1):R10. doi: 10.1186/gb-2010-11-1-r10 (PMC2847713; doi:10.1186/gb-2010-11-1-r10)
Supplement: Additional file 5 — Comparison of observed X-linked delta values to TA/EX values using a Wilcoxon test for the Yoruba-French and French-Han population pairs. [file gb-2010-11-1-r10-S5.doc]

**A.**

**B.**

**Figure S3: Comparison of TA/EX and Observed X-Linked Delta Values.** A) The female proportion of the effective population size and the female proportion of migration were both varied over a range from 0.01 to 0.99. For each of the 9,800 possible pairs of these values, a list of TA/EX values was produced from the observed Yoruba-French autosomal delta values. This list of TA/EX values was compared to the list of observed X-linked delta values using a two-sided Wilcoxon Test. The color at a given point represents the resulting p-value of this comparison. Red locations on the grid represent N_f_/N, m_f_/m value-pairs that produce sets of TA/EX delta values that significantly differ from the observed X-linked values; white and yellow regions on the grid represent value-pairs that produce TA/EX values that do not significantly differ from the observed X-linked values. B) The same as in A, except that the observed French-Han autosomal delta values were used as input for the transformation.
