# Supplementary material for: Characterization of X-Linked SNP genotypic variation in globally distributed human populations
Source: Genome Biol. 2010 Jan 28;11(1):R10. doi: 10.1186/gb-2010-11-1-r10 (PMC2847713; doi:10.1186/gb-2010-11-1-r10)
Supplement: Additional file 6 — Number of high Fst SNPs and regions contained in various sets of TA/EX values and comparison of observed X-linked Fst values to various TA/EX Fst values using a Wilcoxon test for all three population pairs. [file gb-2010-11-1-r10-S6.doc]

**A. B.**

**C.**

**D. E.**

**F.**

**G. H.**

**I.**

**Figure S4: Delta Analyses Repeated using Pairwise Fst Values.** The pairwise Fst values for all autosomal and X-linked SNPs were calculated for the Yoruba-Han, Yoruba-French, and French-Han population pairs. For a particular population pair, SNPs with Fst > 0.9 were classified as high Fst SNPs. A) The female proportion of the effective population size and the female proportion of migration were both varied over a range from 0.01 to 0.99. For each of the 9,800 possible pairs of these values, a list of TA/EX values was produced from the observed autosomal Fst values for the Yoruba-Han. The color at a given point on the grid represents the number of high Fst SNPs out of 640,698 total SNPs found in this list. B) The same as in A, except that the color at a given point on the grid signifies the number of high Fst regions out of 13,395 total regions represented by each list of TA/EX Fst values. C) The female proportion of the effective population size and the female proportion of migration were both varied over a range from 0.01 to 0.99. For each of the 9,800 possible pairs of these values, a list of TA/EX values was produced from the observed autosomal Fst values for the Yoruba-Han. This list of TA/EX values was compared to the list of observed X-linked Fst values using a two-sided Wilcoxon Test. The color at a given point represents the resulting p-value of this comparison. Red locations on the grid represent N_f_/N, m_f_/m value-pairs that produce sets of TA/EX Fst values that significantly differ from the observed X-linked values; white and yellow regions on the grid represent N_f_/N, m_f_/m value-pairs that produce TA/EX values that do not significantly differ from the observed X-linked values. D,E,F) The same as in A,B,C, except that the observed Yoruba-French autosomal Fst values were used as input for the transformation. G,H,I) As with delta, there were no X-linked SNPs with Fst values exceeding 0.9 for the French-Han population pair. Because of this, we produced figures G and H by tabulating the number of SNPs and regions, respectively, for which Fst exceeded 0.8. Otherwise, G, H, and I, are the same as in A, B, C, except that the observed French-Han autosomal Fst values were used as input for the transformation.

The above results are similar to those observed when the same analyses were carried out with delta values rather than pairwise Fst values, except that the N_f_/N, m_f_/m value-pairs that produce TA/EX Fst values that do not differ significantly from the observed X-linked values are shifted somewhat down and to the left on the N_f_/N, m_f_/m value grid. Because of this, it is difficult to ascertain from the above figures whether there is an excess of high Fst SNPs on the X chromosome, especially for the Yoruba-French and French-Han population pairs. To determine whether this excess exists for pairwise Fst values or not, we compared observed X-linked Fst values to TA/EX values produced using selected values of N_f_/N, m_f_/m (see Table S2 for a detailed description of this analysis and its results). This analysis confirmed the presence of an excess of high Fst SNPs and regions on the X chromosome for the Yoruba-Han and Yoruba-French pairs and of an excess of X-linked SNPs with Fst > 0.8 for the French-Han population pair.
