# Supplementary material for: Characterization of X-Linked SNP genotypic variation in globally distributed human populations
Source: Genome Biol. 2010 Jan 28;11(1):R10. doi: 10.1186/gb-2010-11-1-r10 (PMC2847713; doi:10.1186/gb-2010-11-1-r10)
Supplement: Additional file 7 — Results of one-sided Wilcoxon tests comparing specific sets of TA/EX Fst values to observed X-linked Fst values. [file gb-2010-11-1-r10-S7.doc]

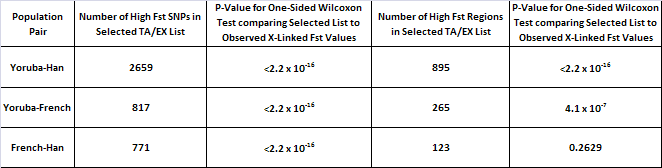


**Table S2: Comparison of Selected TA/EX Fst Values to Observed X-Linked** **Fst Values.** For each of the three population pairs, we calculated the number of X-linked high Fst SNPs and regions we observed out of 640,698 and 13,395, respectively. These values are given in Figure S4. Out of all the lists of TA/EX Fst values produced by varying N_f_/N and m_f_/m from 0.01 to 0.99, we selected one list that contained a similar number of high Fst SNPs or regions (to make this analysis more conservative, we actually always selected a list that contained proportionally fewer high Fst SNPs or regions than was observed for the X chromosome; see the above table for the number of high Fst SNPs or regions in each selected list). We then used a one-sided Wilcoxon test to assess whether the selected TA/EX values were greater than the observed X chromosome Fst values. The p-values for these tests are shown in the above table. For all tests except one (the one involving regions for the French-Han population pair), we reject the null hypothesis with a very small p-value. This suggests that while the selected lists of TA/EX values and observed X-linked Fst values have similar numbers of high Fst SNPs and regions, the TA/EX values are overall high enough for the distributions of the two lists to be significantly different. This, in turn, suggests that lists of TA/EX Fst values representing proportionally fewer high Fst SNPs or regions would be more similar in overall distribution to the observed X-linked Fst values.
