# Supplementary material for: Characterization of X-Linked SNP genotypic variation in globally distributed human populations
Source: Genome Biol. 2010 Jan 28;11(1):R10. doi: 10.1186/gb-2010-11-1-r10 (PMC2847713; doi:10.1186/gb-2010-11-1-r10)
Supplement: Additional file 8 — Genic enrichment (relative to the ratio of genic SNPs to all SNPs) of SNPs in different delta bins for all three population pairs. [file gb-2010-11-1-r10-S8.doc]

A.


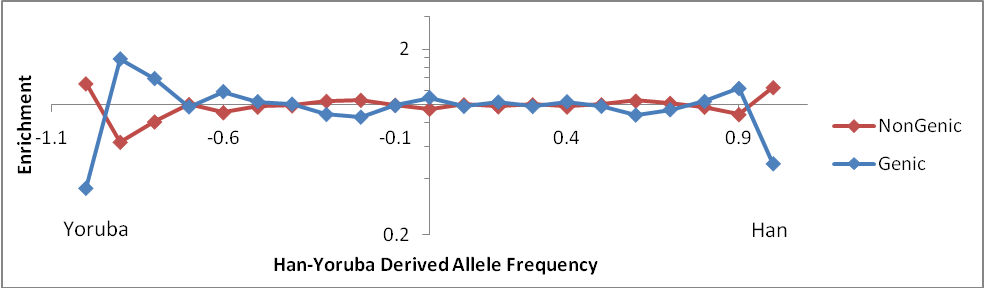


B.


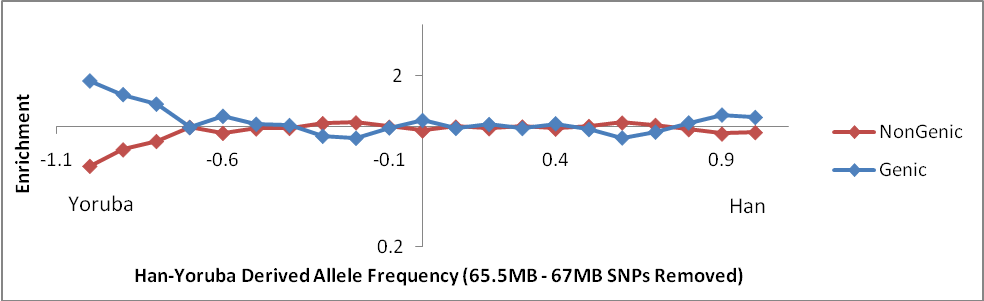


C.


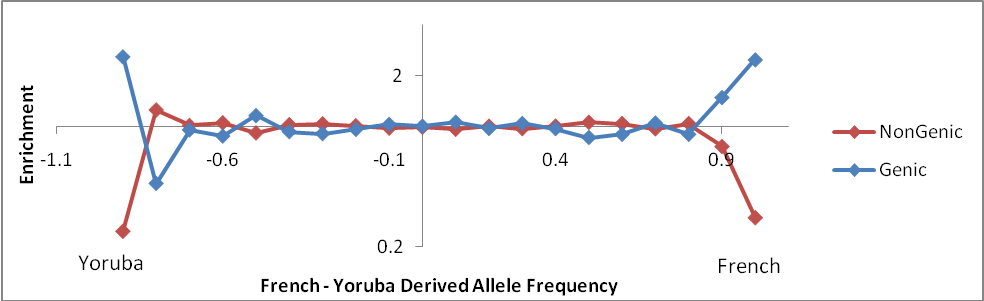


D.


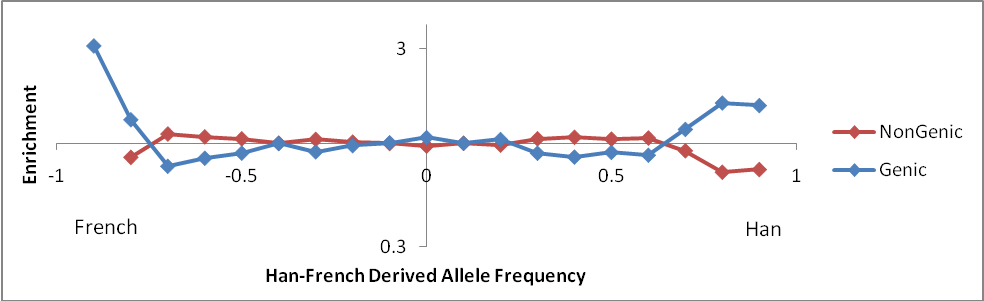


**Figure S5: Enrichment of genic SNPs among SNPs with large delta values.** SNPs were grouped into bins based on the value of their derived allele frequency in the first population minus their derived allele frequency in the second population. Each data point represents a bin. The y-value of each genic data point was obtained by dividing the fraction of genic SNPs in the corresponding bin by the overall fraction of genic SNPs. The y-value of each non-genic data point was then obtained by the same procedure, substituting the bin and overall fractions of non-genic SNPs for the fractions of genic SNPs. A, C, D) These plots were created using all X chromosome SNPs for which ancestral/derived allele information could be obtained. B) This plot was created using all X chromosome SNPs with ancestral/derived allele information, except those that fell between 65.5MB and 67MB (this includes all the SNPs in the largest Yoruba-Han high-delta region).
