# Supplementary material for: Characterization of X-Linked SNP genotypic variation in globally distributed human populations
Source: Genome Biol. 2010 Jan 28;11(1):R10. doi: 10.1186/gb-2010-11-1-r10 (PMC2847713; doi:10.1186/gb-2010-11-1-r10)
Supplement: Additional file 9 — Sample sets used for different iHS, CLR, and XP-EHH runs. [file gb-2010-11-1-r10-S9.doc]

**Note S2**

**Tests of Selection**

For brevity, only 3 sets of test scores – one iHS, one CLR, and one XP-EHH - were discussed in the main text [18,19,20]. However, in the course of our work, we calculated additional sets of CLR and XP-EHH scores. All rounds of iHS, CLR, and XP-EHH testing are outlined below and the results from all tests are given in Table S3. As discussed in the text, we divided the CEPH-HGDP populations into a total of 8 continental groups – African Agriculturists, Hunter-Gatherers, Middle Easterners, Europeans, Central Asians, East Asians, Oceanians, and Americans. For each of the 8 continental groups, we also selected one representative population - Biaka, Yoruba, Palestinian, French, Burusho, Han, Papuan, and Maya – with the intention of running each of the three tests on both the 8 continental sample sets and on these 8 representative populations. Ultimately, we elected to calculate iHS scores only for the continental groups, as iHS does not perform well with small samples [10]. We did, however, calculate CLR scores for both continental groups and representative populations; the resulting scores were similar for the two types of sample set (see Table S3). We calculated a total of 6 sets of XP-EHH scores. The XP-EHH test requires as input both a reference and a study sample set as it compares haplotype structure in the study set to the reference set to produce the resulting stores. We selected 3 continents from our set of 8 to act as reference sample sets – African Agriculturists, Europeans, and East Asians. The first round of XP-EHH scores used African Agriculturists as the reference sample and the other 7 continents as study samples; the second and third rounds used Europeans and East Asians as the reference samples, respectively. Three more sets of XP-EHH scores were then calculated using representative populations. The first of these used Yoruba as the reference sample and the other 7 representative populations as study samples; the second and third used the French and the Han as the reference samples, respectively. After calculating the last set of XP-EHH scores, 5 continents – Hunter-Gatherers, Middle Easterners, Central Asians, Oceanians, and Americans – had a total of 9 test scores associated with each chromosomal region – one iHS, two CLR, and six XP-EHH scores. The remaining three continents – African Agriculturists, Europeans, and East Asians – (and their representative populations) were each used as a reference for two sets of XP-EHH testing and had only 7 scores each – one iHS, two CLR, and four XP-EHH scores.

For the main text, we focused on one set of iHS scores, one set of CLR scores, and one set of XP-EHH scores. From the two available sets of CLR scores, we selected the one calculated using continental groups to increase our ability to directly compare iHS and CLR results (as the only set of iHS scores were calculated using continental groups). For the same reason, we wanted to select a set of XP-EHH scores calculated using continental groups. This still left us with two or three possible XP-EHH scores for each continent (since we calculated XP-EHH scores using three difference reference continents), so we next took into consideration the relationship between the reference and study sample sets. Selective sweeps often encompass multiple neighboring continents. However, because XP-EHH compares haplotype structure in the study sample to that in the reference sample, XP-EHH is only elevated in chromosomal regions that are under selection in the study sample but not the reference sample. Because of this, XP-EHH is most sensitive to sweeps when the reference sample and study sample are genetically distant from one another. As a result, for the two African continental groups, we chose to include in the main text XP-EHH scores that were calculated using East Asians as the reference sample (and African Agriculturists and Hunter-Gatherers as the study samples). For all non-African continental groups, we included in the main text XP-EHH scores that were calculated using African Agriculturists as the reference sample (and each of the six non-African continents as the study samples). The double line separating the African Agriculturists from the Middle Easterners in the XP-EHH portion of Figure 3 indicates that the XP-EHH scores represented on the left side of the lines where calculated using a different reference sample than those represented on the right side of the lines.
