# Supplementary material for: Characterization of X-Linked SNP genotypic variation in globally distributed human populations
Source: Genome Biol. 2010 Jan 28;11(1):R10. doi: 10.1186/gb-2010-11-1-r10 (PMC2847713; doi:10.1186/gb-2010-11-1-r10)
Supplement: Additional file 10 — Raw iHS, CLR, and XP-EHH scores for all 372 X chromosome regions and all 8 continental groups. [file gb-2010-11-1-r10-S10.doc]

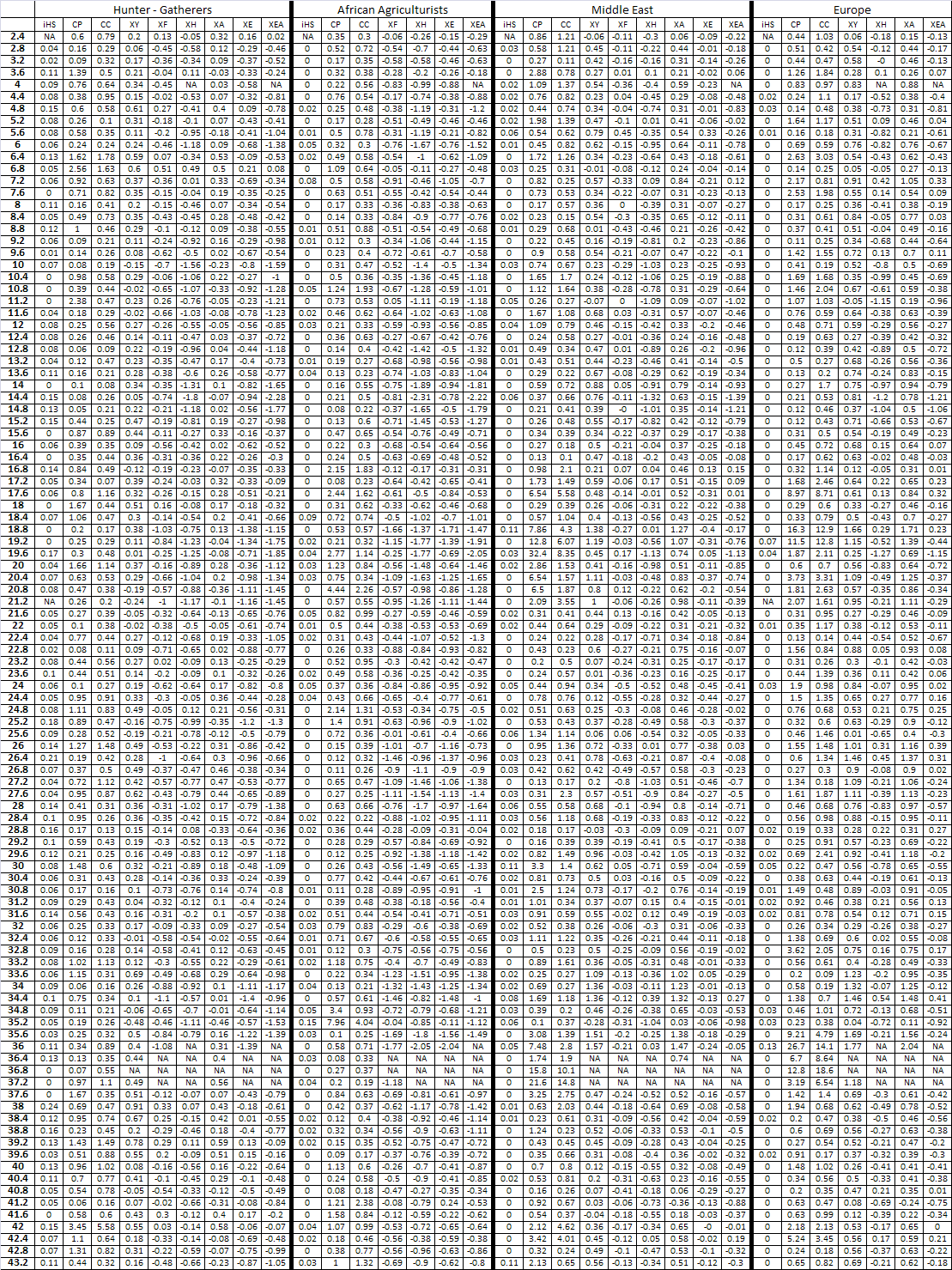


**Table S3**


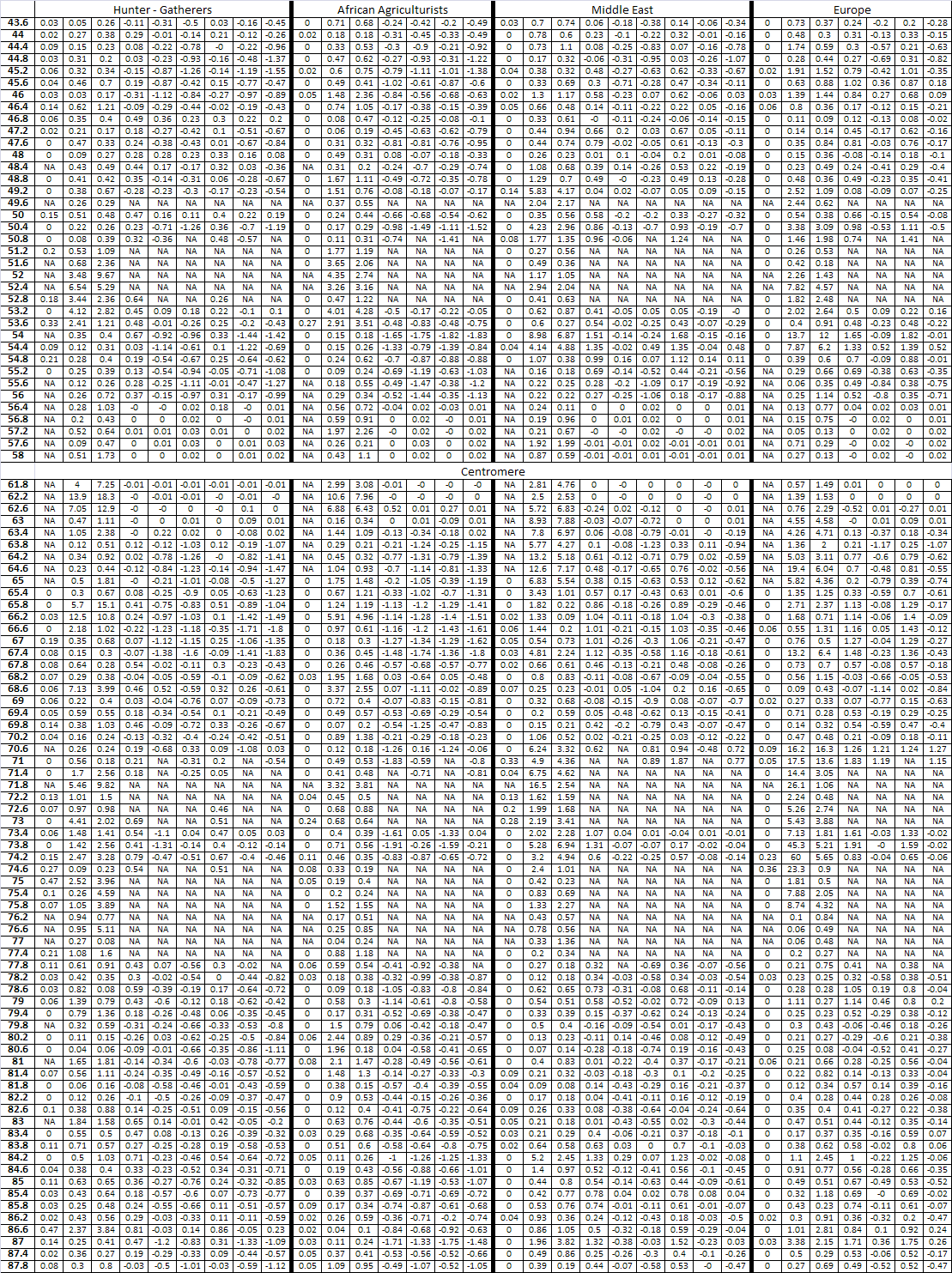


**Table S3, Continued**


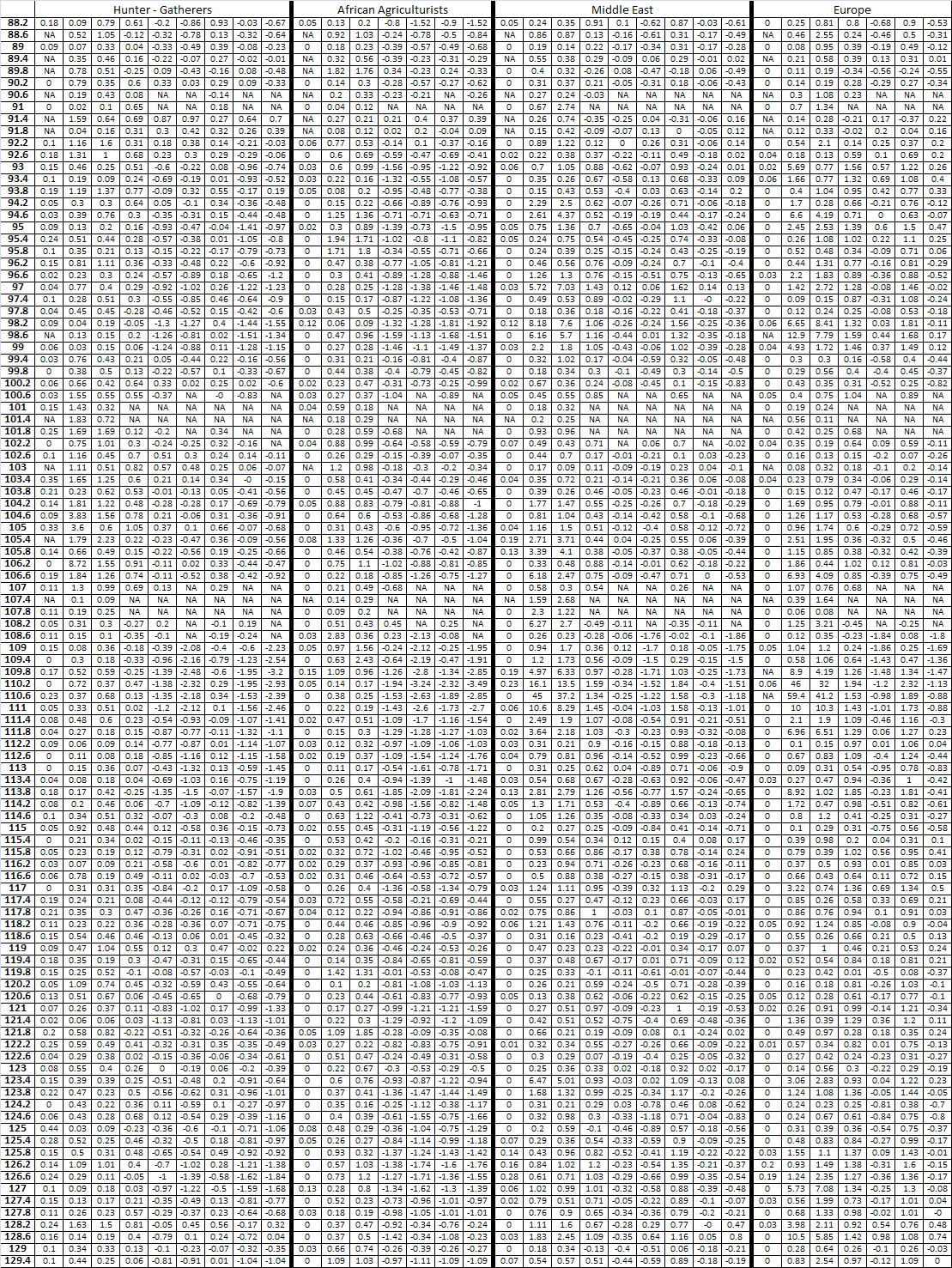


**Table S3, Continued**


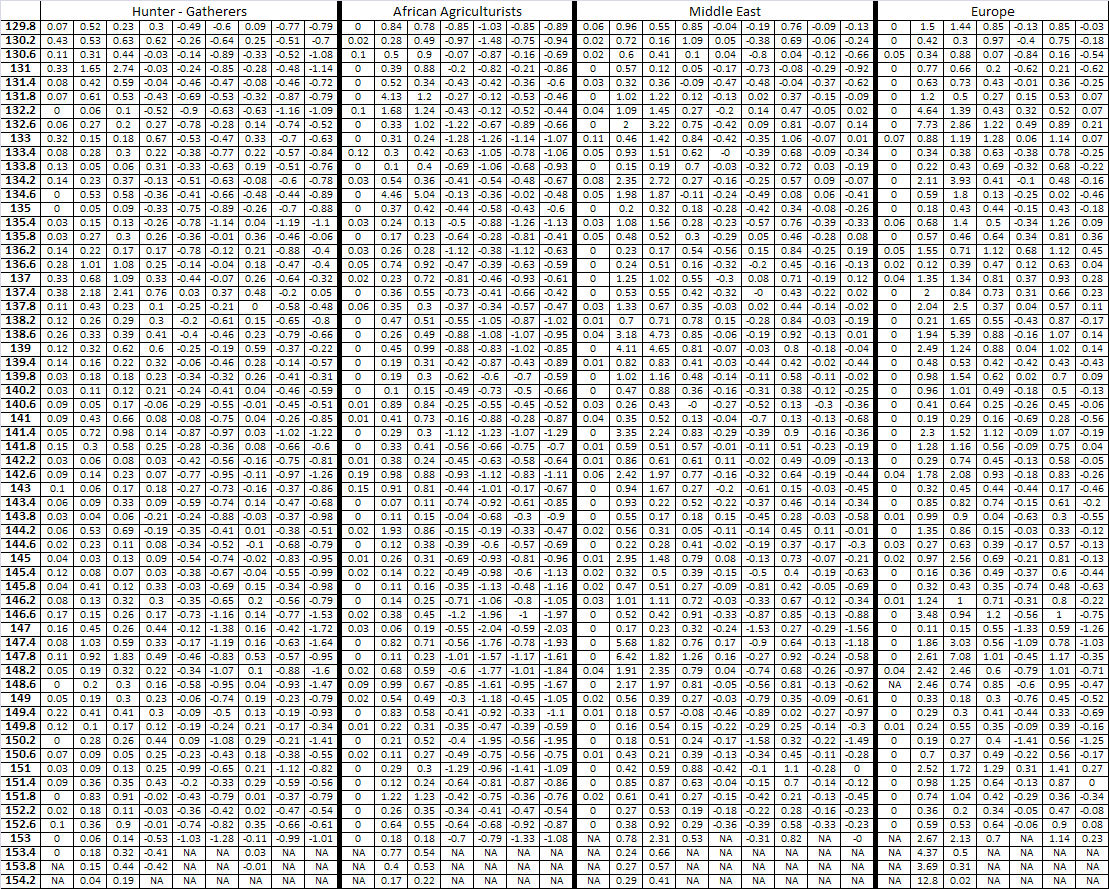


**Table S3, Continued**


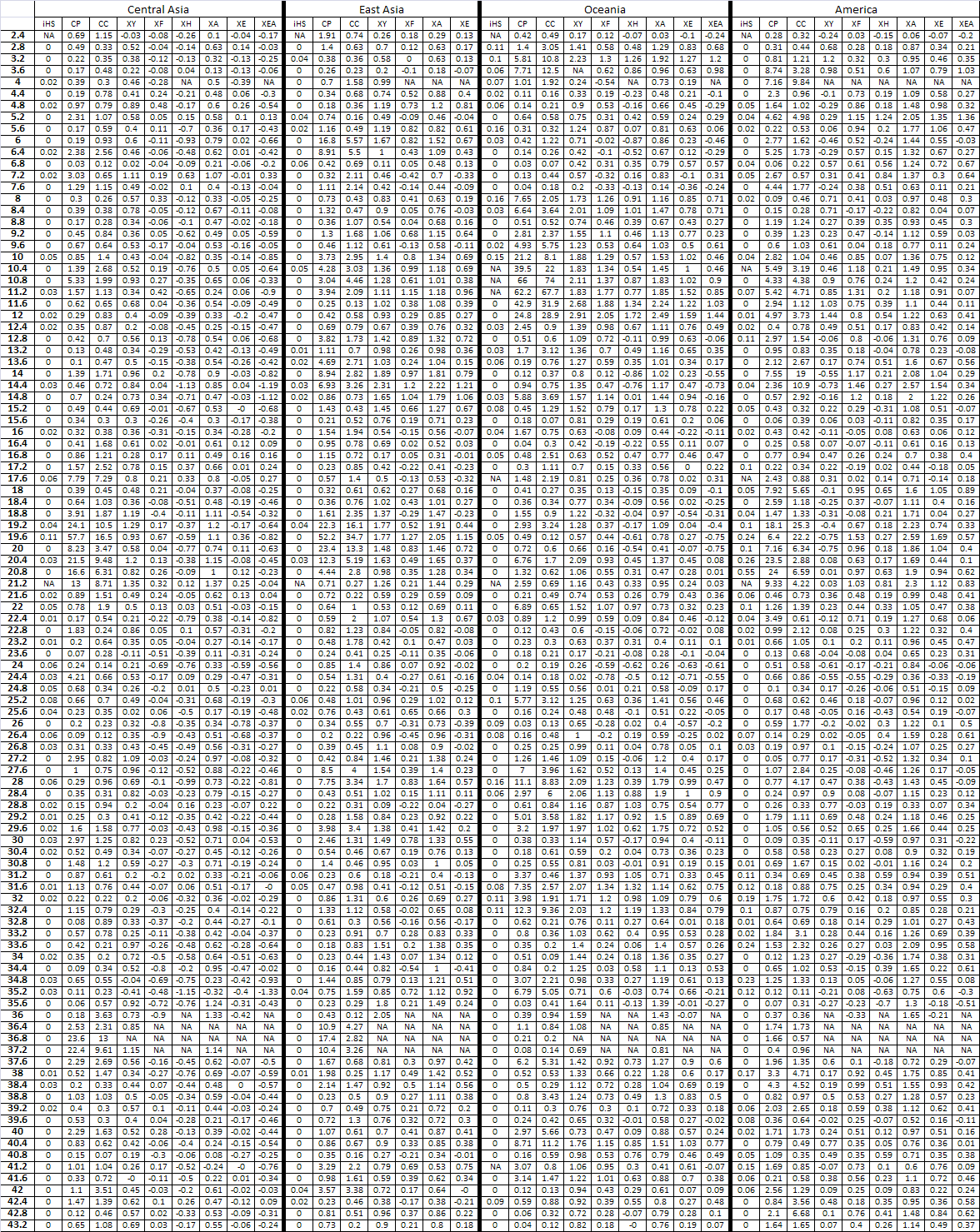


**Table S3, Continued**


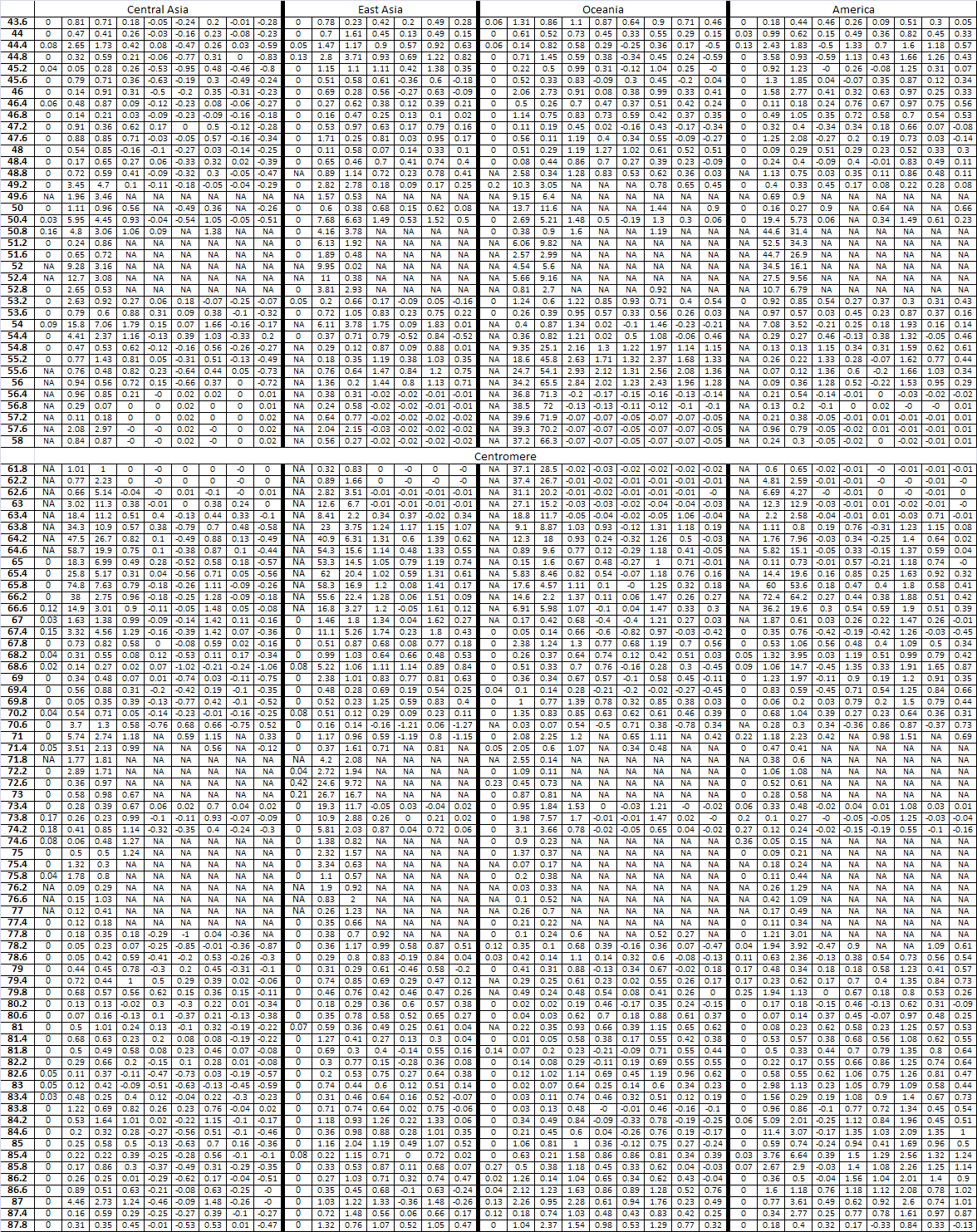


**Table S3, Continued**


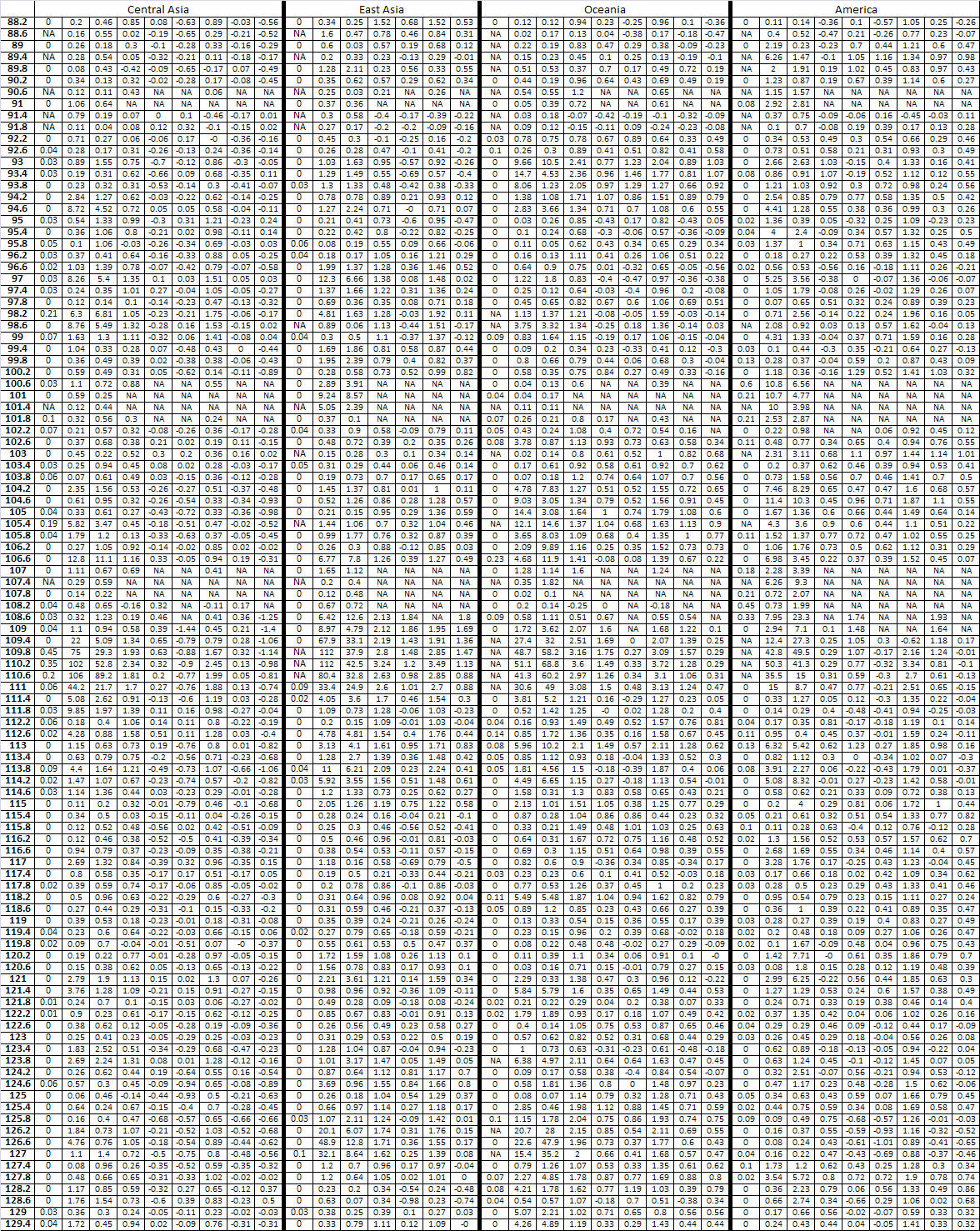


**Table S3, Continued**


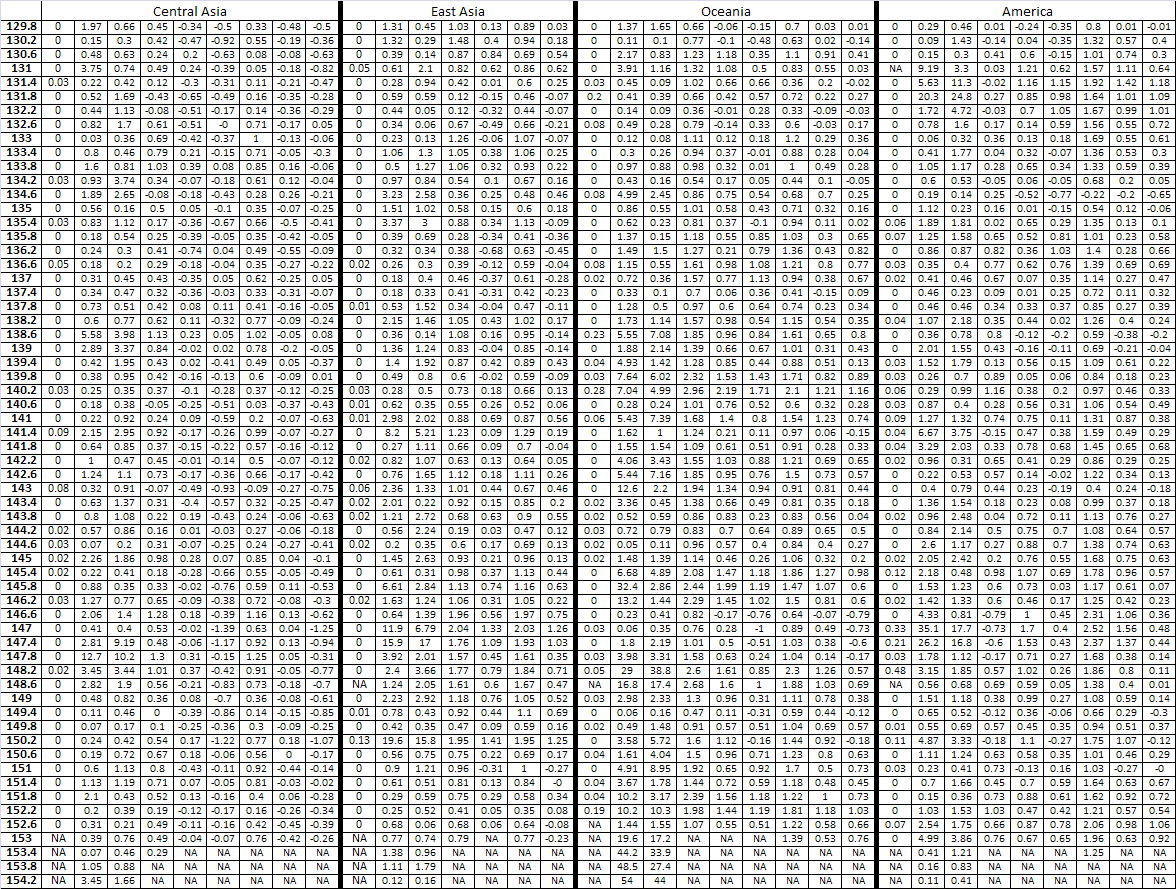


**Table S3: Complete Results of Tests of Selection.** Each row represents a 400KB region beginning at the indicated chromosomal position (given in MB). Individual columns represent a particular test of selection (these columns are labeled at the top of pages 1 and 5, just below the continental group labels; CP – CLR with populations, CC – CLR with continents, XY – XPEHH with Yoruba Reference, XF – XPEHH with French Reference, XH – XPEHH with Han Reference, XA – XPEHH with African Agriculturist Reference, XE – XPEHH with European Reference, XEA – XPEHH with East Asian Reference). Each large column represents a continental group. All continental groups have either 7 or 9 test columns.
